# Dietary Periodicity Disrupts the Gut Microbiota–Enterolactone Axis to Exacerbate MASLD in a Translational Guinea Pig Model

**DOI:** 10.3390/nu18152573

**Published:** 2026-08-06

**Authors:** Xiaoli Zhang, Yusha Li, Rongping Luo, Haiyun Wang, Jing Guo, Xiaohan Zhang, Manish Kumar, Yi Li, Jing Liu

**Affiliations:** 1 China-New Zealand Joint Laboratory on Biomedicine and Health, Guangzhou Institutes of Biomedicine and Health, Chinese Academy of Sciences, Guangzhou 510530, China; zhang_xiaoli01@gibh.ac.cn (X.Z.);; 2 Institute of Development and Regeneration, Guangzhou Institutes of Biomedicine and Health, Chinese Academy of Sciences, Guangzhou 510530, China; 3 Guangdong Provincial Key Laboratory of Stem Cell and Regenerative Medicine, Guangdong-Hong Kong Joint Laboratory for Stem Cell and Regenerative Medicine, Guangzhou Institutes of Biomedicine and Health, Chinese Academy of Sciences, Guangzhou 510530, China; 4 Centre for Regenerative Medicine and Health, Hong Kong Institute of Science & Innovation, Chinese Academy of Sciences, Hong Kong SAR, China; 5 Analytical Instrumentation Core, Guangzhou Institutes of Biomedicine and Health, Chinese Academy of Sciences, Guangzhou 510530, China; 6 Joint School of Life Sciences, Guangzhou Institutes of Biomedicine and Health, Chinese Academy of Sciences, Guangzhou Medical University, Guangzhou 510530, China

**Keywords:** gut microbiota–metabolite axis, intermittent high-fat high-cholesterol diet, liver injury, flaxseed lignan supplementation

## Abstract

**Background/Objectives:** Metabolic dysfunction-associated steatotic liver disease (MASLD) is closely linked to Western dietary patterns. Yet, preclinical studies rely on continuous high-fat feeding, overlooking the intermittent nature of human eating. Whether dietary periodicity itself influences the gut–liver axis and MASLD pathogenesis remains unknown. We compared continuous and intermittent high-fat, high-cholesterol (HFHC) diets in guinea pigs, a model that mirrors human lipoprotein metabolism, hepatic cholesterol handling, and hindgut fermentation. **Methods:** Integrated multi-omics analyses (serum metabolomics, fecal 16S rRNA sequencing, and liver transcriptomics) were employed in a guinea pig model, stratified into normal diet (ND), intermittent HFHC diet (IHD), and IHD with flaxseed lignan supplementation, to compare the effects of dietary regimens and the therapeutic efficacy of lignan on hepatic pathology. **Results:** Both diets induced hallmark hepatic features of MASLD; however, the intermittent regimen provoked significantly more severe hepatic steatosis, inflammation, oxidative stress, and fibrosis. Hepatic transcriptomic analysis identified the PI3K-Akt signaling pathway as the most significantly enriched pathway in the IHD group. Mechanistically, this aggravated liver injury was linked to gut microbiota dysbiosis and a marked depletion of the microbial metabolite enterolactone. Fecal microbiota transplantation confirmed that the dysbiotic microbiota directly transmits the aggravated liver injury phenotype. Supplementation with flaxseed lignan, the dietary precursor of enterolactone, restored enterolactone production, corrected the dysregulated gut microbiota–enterolactone axis, and largely normalized the expression of PI3K-Akt downstream targets, thereby alleviating hepatic pathology. **Conclusions:** These findings suggest that alterations in the gut microbiota–enterolactone axis may contribute to diet-periodicity-driven liver injury and highlight its potential involvement in disease progression. Modulating this axis through dietary interventions, such as flaxseed lignan supplementation, may represent a promising nutritional strategy for mitigating MASLD associated with cyclical dietary exposure.

## 1. Introduction

Metabolic dysfunction-associated steatotic liver disease (MASLD) is a global health crisis driven largely by modern dietary habits [1,2,3]. A critical limitation in preclinical research is the near-universal reliance on continuous high-fat feeding paradigms, which, although effective in inducing steatosis, fail to recapitulate the cyclical, intermittent nature of human Western diet consumption. This is particularly relevant given that populations with cyclical dietary exposure can develop MASLD even in the absence of overt caloric excess, suggesting that dietary periodicity is an independent contributor to disease risk. The physiological consequence of this mismatch is underscored by cardiovascular research demonstrating that intermittent, but not continuous, hyperlipidemia accelerates atherosclerosis through lasting reprogramming of resident macrophages and neutrophils [4,5]. Together, these observations establish dietary periodicity as an independent pathological factor and underscore the urgent need to adopt dynamic feeding models in MASLD research. To date, however, a systematic comparison of continuous versus intermittent high-fat, high-cholesterol (HFHC) dietary regimens in a translationally relevant MASLD model is entirely lacking.

The guinea pig (*Cavia porcellus*) is uniquely positioned to bridge this gap because it circumvents the fundamental limitations of murine models in recapitulating human lipid metabolism and MASLD pathogenesis. A pivotal species divergence lies in hepatic apolipoprotein B (ApoB) mRNA editing. The mouse liver highly expresses the editing enzyme, which converts ApoB-100 to the truncated ApoB-48. Consequently, mice secrete ApoB-48-containing VLDL particles that are rapidly cleared and cannot be converted to LDL, yielding an HDL-dominant profile and marked resistance to dietary cholesterol-induced hypercholesterolemia [6]. In stark contrast, the guinea pig—like the human—exhibits negligible hepatic ApoB mRNA editing, produces ApoB-100-containing VLDL, and maintains an LDL-dominant lipoprotein profile that is exquisitely sensitive to dietary fat and cholesterol [7,8,9,10,11,12]. This fundamental similarity extends to hepatic cholesterol handling: upon dietary cholesterol challenge, wild-type mice partition excess cholesterol into bile acids for excretion, whereas guinea pigs and humans accumulate hepatic cholesteryl ester, a hallmark of human MASLD that directly contributes to lipotoxicity, inflammasome activation, and stellate cell-driven fibrogenesis. Because of these metabolic defenses, mice typically require genetic modifications (e.g., Ldlr^−/−^ or Apoe^−/−^) or choline-deficient diets to develop robust steatohepatitis with fibrosis: interventions that distort the very metabolic pathways they aim to model [13]. In contrast, the guinea pig fed a high-fat, high-cholesterol diet spontaneously progresses from steatosis to steatohepatitis with ballooning degeneration and bridging fibrosis, faithfully recapitulating the histological spectrum of human disease without genetic or severe nutritional manipulation [11]. Beyond hepatic lipid metabolism, the guinea pig offers a critical advantage in modeling the gut–liver axis. As a strict herbivore adapted to a high-fiber diet, it possesses a voluminous, fermentation-competent cecum that generates short-chain fatty acids and other microbiota-derived metabolites in a pattern that closely mirrors human colonic fermentation, a feature that the murine cecum—given the mouse’s omnivorous physiology—replicates less faithfully [14,15,16]. This unique confluence of human-like lipoprotein handling, dietary-driven steatohepatitis, and hindgut fermentation establishes the guinea pig as a superior translational platform for investigating how diet composition and pattern intersect to drive MASLD.

To address the identified gap, we performed an integrated multi-omics analysis combining serum metabolomics, fecal 16S rRNA sequencing, and liver transcriptomics in guinea pigs subjected to continuous versus intermittent HFHC feeding. We further tested a targeted nutritional intervention—dietary flaxseed lignan supplementation—to determine whether restoration of the gut microbiota–enterolactone axis can counteract the liver injury specifically driven by the intermittent regimen.

## 2. Materials and Methods

### 2.1. Animals

One-year-old Dunkin Hartley guinea pigs (Beijing Vital River Laboratory Animal Technology Co., Ltd., Beijing, China) were acclimated for one week, then housed in environmentally controlled cages (optimal temperature, 12 h light/dark cycles) with unrestricted access to food and water. The study was conducted according to applicable animal research and institutional welfare guidelines, and approved by the Institutional Review Board of the Guangzhou Institutes of Biomedicine and Health (GIBH), Chinese Academy of Sciences (protocol code 2025094; approval date: 30 September 2025).

### 2.2. Study Design

**Figure 1:**
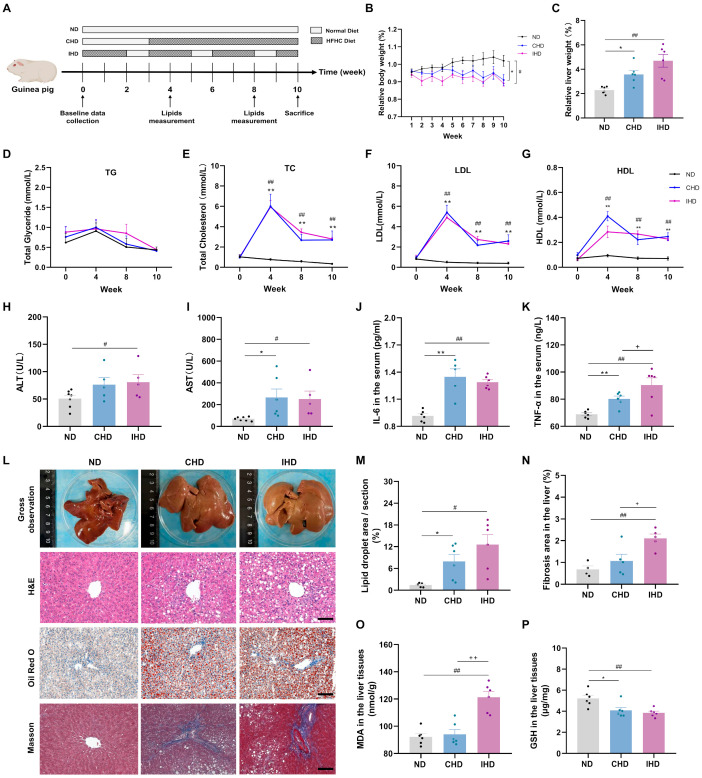
Comparison of the effects of continuous and intermittent HFHC diet on physiological characteristics, liver function and hepatic histomorphology in guinea pigs. (**A**) Schematic of the experimental design. (**B**) Body weight. (**C**) Relative liver weight. (**D**–**G**) Blood lipid levels: (**D**) TG, (**E**) TC, (**F**) LDL, (**G**) HDL. (**H**) ALT level; (**I**) AST level; (**J**) Serum IL-6 level; (**K**) Serum TNF-α level; (**L**) representative liver morphology (gross appearance, H&E, Oil Red O, and Masson staining). Scale bar, 100 µm; original magnification, ×200. (**M**) Hepatic lipid droplet area; (**N**) hepatic fibrosis area; (**O**) MDA level in liver tissue; (**P**) GSH level in liver tissue. Data are presented as mean ± SEM (*n* = 5–6 per group). Body weight and serum lipid concentrations measured at different time points were analyzed using a linear mixed-effects model. For other data, one-way ANOVA followed by a multiple comparison test was used for statistical analysis. * *p* < 0.05, ** *p* < 0.01 (CHD vs. ND); # *p* < 0.05, ## *p* < 0.01 (IHD vs. ND); + *p* < 0.05, ++ *p* < 0.01 (IHD vs. CHD).

Eighteen Dunkin Hartley guinea pigs were randomly assigned to three groups (*n* = 6/group) for a 10-week feeding study: normal diet (ND, standard chow), continuous HFHC diet (CHD, standard chow for 3 weeks then HFHC diet from week 4 to 10; 89.5% standard chow, 10% fat, 0.5% cholesterol), and intermittent HFHC diet (IHD, two weeks HFHC followed by one week standard chow, cycled until week 10). Diets were from Tianjin Ke’ao Xieli Feed Co., Ltd., Tianjin, China, based on a published protocol [7]. Experimental design is shown in Figure 1A. Body weight was recorded weekly; food intake was recorded daily. Blood was collected from the orbital sinus at baseline, weeks 4 and 8. Fecal samples were collected before termination and stored at −80 °C. At week 10, animals were anesthetized with isoflurane (3–5% induction, 1.5–2.5% maintenance, 1.0–1.5 L/min O_2_) and euthanized by exsanguination via cardiac puncture under deep anesthesia. Blood and liver tissues were collected for subsequent analyses.

### 2.3. Antibiotic Treatment and Fecal Microbiota Transplantation

Antibiotic (Abx) treatment was performed as described previously [17]. One-year-old guinea pigs (~800 g) received a daily oral gavage for 7 days of an antibiotic cocktail (ampicillin 100 mg/mL, neomycin 100 mg/mL, metronidazole 100 mg/mL, vancomycin 50 mg/mL) at 2 µL/g body weight. Post-treatment, fecal samples were collected for microbiome sequencing to confirm gut microbiota depletion. For fecal microbiota transplantation (FMT), fresh feces were collected from CHD or IHD donors after the 10-week feeding period. Antibiotic-pretreated animals were divided into three groups (*n* = 3 each): saline control, FMT-CHD, and FMT-IHD. Donor feces (1 g) were suspended in 4 mL sterile saline, sequentially filtered through 80, 200, and 400 mesh gauze, and centrifuged (1500× *g*, 10 min) to obtain clarified suspension. Recipients received daily oral gavage of the corresponding suspension (3.5 mL/kg) on a normal diet for 18 days. Afterwards, feces, blood, and liver tissues were collected.

### 2.4. Design of Flaxseed Lignan Extract Intervention

One-year-old guinea pigs were randomly assigned to three groups: (1) Control (ND, *n* = 3); (2) IHD (HFHC diet cycled as before, saline gavage, *n* = 4); and (3) IHD + Lignan (*n* = 7), receiving daily oral gavage of a commercial flaxseed lignan extract (Shanxi Jiuzhou Kangyuan Biotechnology Co., Ltd., Xi’an, China) standardized to contain 40% secoisolariciresinol diglucoside (SDG). According to the manufacturer, the remaining 60% consists primarily of maltodextrin (carrier) and other uncharacterized flaxseed-derived components, which may include minor lignans (e.g., secoisolariciresinol, matairesinol), dietary fiber, and residual protein. The extract was administered at 0.5 g/kg body weight, equivalent to 200 mg SDG/kg BW, a dose shown to be well tolerated in preliminary observations. This intervention tests the enterolactone-producing pathway by providing SDG, a dietary lignan precursor metabolized by the gut microbiota [18,19]. Blood lipids were measured at baseline, week 4, and week 8. After 10 weeks, fecal, blood, and liver tissues were collected.

### 2.5. Measurement of Serum and Hepatic Biochemical Parameters

Fasting blood glucose was measured using a glucometer (Sinocare, Changsha, China) during blood sample collection. Blood samples were centrifuged at 1000× *g* for 10 min to obtain serum. Serum levels of total cholesterol (TC), triglycerides (TG), high-density lipoprotein (HDL), low-density lipoprotein (LDL), alanine aminotransferase (ALT), aspartate aminotransferase (AST), blood urea nitrogen (BUN), and creatinine were analyzed using a Hitachi 7020 automated biochemical analyzer (Hitachi High-Technologies, Tokyo, Japan). Serum interleukin-6 (IL-6) (Cat No. MM-035101) and tumor necrosis factor-α (TNF-α) (Cat No. MM-033701) were measured with enzyme-linked immunosorbent assay (ELISA) kits (Jiangsu Meimian Industrial Co., Ltd., Yancheng, China). Serum insulin was measured by an ELISA kit (Cat No. JL21557, Jianglai Biotechnology Co., Ltd., Shanghai, China). The homeostasis model assessment of insulin resistance (HOMA-IR) was calculated according to a previously published method [20]. Hepatic levels of malondialdehyde (MDA) (Cat No. JM-07030G1), and glutathione (GSH) (Cat No. JM-07067G1) were determined using corresponding ELISA kits (Jiangsu Jingmei Biotechnology Co., Ltd., Yancheng, China) according to the manufacturer’s instructions. Serum total enterolactone levels were determined using a commercially available ELISA kit (MyBioSource, Cat. No. MBS7270528, San Diego, CA, USA). Prior to analysis, serum samples were treated with β-glucuronidase to hydrolyze conjugated enterolactone, allowing measurement of total enterolactone (free plus glucuronide-conjugated forms).

### 2.6. Liver Morphology

Liver samples for hematoxylin and eosin (H&E) and Masson’s trichrome staining were rinsed with PBS, fixed in 4% paraformaldehyde, and embedded in paraffin. Sections (3 μm) were cut using a microtome and stained with H&E for assessment of lipid droplets and inflammation, and with Masson’s trichrome for evaluation of fibrosis. For Oil Red O staining, fresh liver tissues were embedded in optimal cutting temperature (OCT) compound. Cryosections (8–10 μm) were cut using a cryostat, air-dried, fixed in 10% neutral buffered formalin for 10 min, rinsed with distilled water, and incubated in 60% isopropanol for 5 min. Sections were then stained with a 0.3% Oil Red O working solution for 15 min at room temperature, differentiated in 60% isopropanol, rinsed in distilled water, and counterstained with hematoxylin. After a final rinse, sections were mounted with aqueous mounting medium and immediately imaged. For each stain, 30 images per slide were captured at 200× magnification (TissueFAXS scanner, Vienna, Austria) and analyzed with ImageJ (version 1.53k, NIH, Bethesda, MD, USA).

### 2.7. Cell Culture and Experimental Procedure

Human liver hepatocellular carcinoma cells (HepG2) were kindly supplied by Dr. Yingying Xu (Guangzhou Institute of Biomedicine and Health, Chinese Academy of Sciences, China). The cells were maintained in Dulbecco’s Modified Eagle Medium (DMEM) supplemented with 10% fetal bovine serum (FBS) and 1% antibiotic/antimycotic (penicillin/streptomycin) at 37 °C in a humidified atmosphere of 5% CO_2_. Cells were passaged upon reaching 70–80% confluency, and the medium was replaced every 48 h.

Before treatment, cells were serum-starved for 3 h in serum-free DMEM. Palmitic acid (PA) (Cat No. P5585-10G, Sigma-Aldrich, Burlington, MA, USA) was dissolved in a mixture of ethanol and 1 M NaOH, heated to 70 °C, mixed with fatty-acid-free bovine serum albumin (2% BSA) and diluted in DMEM as it was described previously [21]. Enterolactone (ENL) (Cat No. 445199-5MG-F, Sigma-Aldrich, USA) was dissolved in DMSO as a stock solution and stored at −20 °C. The cells were divided into five groups: (1) control group, incubated with DMEM containing 2% BSA and an equivalent volume of DMSO (vehicle control); (2) PA group, treated with 0.5 mM PA; (3–5) PA + ENL groups, co-treated with 0.5 mM PA and 10 μM, 30 μM, or 50 μM ENL, respectively. All treatments lasted for 24 h.

After the incubation, cell viability was assessed using a CCK-8 kit (Cat No. CK04-100T, DOJINDO, Kumamoto, Japan) according to the manufacturer’s protocol. Intracellular reactive oxygen species (ROS) were measured with a ROS assay kit (Cat No. S0033S, Beyotime, Shanghai, China). Triglyceride (TG) and total cholesterol (TC) contents were quantified using commercial kits (Cat No. E1013-105 and E1015-105, Applygen, Beijing, China). For Oil Red O staining, cells were washed with PBS, fixed in 4% paraformaldehyde for 30 min, rinsed with 60% isopropanol, and stained with freshly prepared Oil Red O working solution for 20 min at room temperature. After rinsing with 60% isopropanol and distilled water, the stained cells were visualized under a light microscope (Leica Microsystems, Wetzlar, Germany). In addition, total RNA was extracted from the cells, and the expression of target genes was detected by qRT-PCR. All experiments were performed independently at least three times.

### 2.8. Non-Targeted Metabolomic Analysis

Serum samples were thawed at 4 °C and processed by protein precipitation with ice-cold acetonitrile (1:3, *v*/*v*). After vortexing (5 min, 600× *g*) and centrifugation (20 min, 11,300× *g*, 4 °C), the supernatant was collected for analysis. Quality control (QC) samples were pooled from all samples.

Metabolite profiling was performed using a Waters ACQUITY UPLC I-Class plus/Thermo QE HF system (Waters, Milford, MA, USA). Separation was achieved on an ACQUITY UPLC HSS T3 column (100 mm × 2.1 mm, 1.8 um) with a gradient of 0.1% formic acid in water (A) and acetonitrile (B) at 0.35 mL/min. MS data were acquired in both positive and negative ionization modes under optimized conditions.

Raw data were processed using XCMS v4.5.1 for baseline filtering, peak detection, integration, and retention time correction. Metabolite identification was performed by matching accurate mass, retention time (RT), MS/MS fragmentation patterns, and isotopic distributions against the METLIN, Lipidmaps (v2.3), HMDB, and the in-house LuMet-Animal 3.0 database.

Processed data underwent RSD filtering, missing value imputation, zero-value replacement, score-based filtering, data merging, and log2 transformation prior to statistical analysis. Differential metabolites were defined according to the following criteria: variable importance in projection (VIP) score > 1.0 from the PLS-DA model, false discovery rate (FDR)-adjusted *p*-value < 0.05 (Student’s *t*-test or one-way ANOVA, as appropriate), and |log_2_ fold change| ≥ 3. Multivariate statistical analyses, including PLS-DA, were conducted using MetaboAnalyst (version 5.0, Xia Lab, McGill University, Montreal, QC, Canada). The PLS-DA model was validated by permutation testing (2000 iterations), with model quality assessed by R^2^Y (goodness of fit) and Q^2^ (predictive ability) values; a negative intercept of the Q^2^ regression line was used to confirm the absence of overfitting. Heatmaps, volcano plots, and pathway enrichment analysis were also performed using MetaboAnalyst.

### 2.9. Quantification of 16S rRNA Amplicon Sequencing

Accurate 16S absolute quantification sequencing (OE Biotech Co., Ltd., Shanghai, China) was performed on fecal bacterial DNA extracted using the DNeasy PowerSoil kit. The V3–V4 region of 16S rRNA was amplified with primers (343F/798R) containing barcodes and Illumina adapters. Libraries were sequenced on an Illumina NovaSeq6000 (Illumina, San Diego, CA, USA) (2 × 250 bp, paired-end). Raw reads were processed with cutadapt (version 4.9, Martin, M., Germany) to remove adapters, then denoised, filtered, merged, and checked for chimeras using DADA2 within QIIME2 (2020.11). Representative sequences were taxonomically annotated against the SILVA v138 database using the q2-feature-classifier (version 2024.5, QIIME 2, USA). Alpha diversity was assessed using Chao1 and Shannon indices, and group differences were evaluated with the Wilcoxon rank-sum test (for two-group comparisons) or the Kruskal–Wallis test (for multi-group comparisons). Beta diversity was computed using unweighted UniFrac distance and visualized by principal coordinate analysis (PCoA); statistical significance of community composition differences was tested by permutational multivariate analysis of variance (PERMANOVA). Differential abundance analysis at the ASV and all taxonomic levels (phylum to species) was initially performed using the Wilcoxon rank-sum test (or Student’s *t*-test for normally distributed data), with a significance threshold of *p* < 0.05. To identify key discriminatory taxa, linear discriminant analysis effect size (LEfSe) was further employed, with an LDA score > 2.0 and *p* < 0.05 considered statistically significant. All statistical analyses were performed in QIIME2 and the R environment (version 4.3.2, R Foundation for Statistical Computing, Vienna, Austria).

### 2.10. RNA-Seq and Data Analysis

RNA-seq and bioinformatic analysis (Guangzhou Heqin Biotechnology Co., Ltd., Guangzhou, China) were conducted on liver tissues from ND, CHD, and IHD groups (*n* = 5/group). Total RNA was extracted using TRIzol, treated with DNaseI, assessed for quality (Nanodrop, Thermo Fisher Scientific, Waltham, MA, USA) and integrity (Agilent 2100, Agilent Technologies, Santa Clara, CA, USA), then sequenced on an Illumina NovaSeq 6000 (150 bp paired-end). Raw reads were quality-filtered using fastp (version 0.23.2, Chen, S., China) to obtain clean reads, which were then aligned to the Cavia porcellus reference genome (GCF_034190915.1) with HISAT2. Gene-level read counts were obtained using RSEM (version 1.3.3, University of Wisconsin–Madison, USA), and expression levels were also calculated as FPKM for data exploration. Differential expression analysis was conducted with DESeq2 (version 1.42.1, Bioconductor, USA), which applies median-of-ratios normalization and uses a negative binomial generalized linear model. *p*-values were corrected for multiple testing using the Benjamini–Hochberg procedure. Genes with an adjusted *p*-value < 0.05 and an absolute log_2_ fold change > 1 were considered significantly differentially expressed. Results were visualized using volcano plots and heatmaps. Functional enrichment analyses were performed for Gene Ontology (GO) and the Kyoto Encyclopedia of Genes and Genomes (KEGG).

### 2.11. Real-Time Quantitative PCR

Candidate genes (from RNA-seq and functional relevance) were quantified in liver by real-time qPCR. Total RNA was extracted with Trizol, reverse-transcribed to cDNA (oligo-dT/random primers), and amplified on a Bio-Rad CFX96 (Bio-Rad, Hercules, CA, USA) with SYBR Green using primers listed in Supplementary Table S1. All reactions were in triplicate, and expression was calculated by the 2^−ΔΔCt^ method.

### 2.12. Statistics

Data are means ± SEM unless otherwise stated. Normality and variance homogeneity were assessed by Shapiro–Wilk test. Normality was assessed by the Shapiro–Wilk test, and homogeneity of variances was assessed by Levene’s test. Body weight and serum lipid concentration measured at different time point were analyzed using a linear mixed-effects model with Group, Time, and Group × Time as fixed effects. A significance level of *p* < 0.05 was adopted, and when a significant Group × Time interaction was detected, simple main effects were examined by comparing estimated marginal means with Bonferroni correction. For other multi-group comparisons, one-way ANOVA with Bonferroni post hoc test was used when data met the assumptions of normality and equal variances; otherwise, the Kruskal–Wallis test followed by Dunn’s post hoc test was applied. For two-group comparisons, an unpaired *t*-test was used for normally distributed data, and the Mann–Whitney U test was used for non-normally distributed data. Correlations were evaluated using Pearson’s correlation coefficient for normal data and Spearman’s rank correlation coefficient for non-normal data. *p* < 0.05 was significant. All statistical analyses were performed using GraphPad Prism 10 and SPSS 24.0.

## 3. Results

### 3.1. Effects of Continuous and Intermittent HFHC Diets on Physiological Characteristics in Guinea Pigs

To investigate the effects of continuous (CHD) and intermittent (IHD) HFHC feeding on guinea pig health, a feeding assay was conducted (Figure 1A). CHD animals received a standard diet for three weeks followed by HFHC diet from week 4 to week 10. IHD animals received HFHC diet for two consecutive weeks followed by one week of standard chow, repeated cyclically until week 10. A linear mixed-effects model showed that the main effects of group (F = 3.28, *p* = 0.068) and time (F = 2.26, *p* = 0.059) did not reach statistical significance, whereas the group × time interaction was significant (F = 3.25, *p* = 0.005) for body weight. Post hoc analysis showed that both CHD and IHD groups had significantly lower body weight than the ND group (Figure 1B). A reduction in daily caloric intake was observed in both the CHD and IHD groups. This is consistent with the herbivorous physiology of guinea pigs, which are poorly adapted to energy-dense, high-fat, high-cholesterol diets and consequently develop sustained hypophagia when transitioned from a high-fiber chow to the HFHC diet. Relative liver weight was significantly increased in both diet groups, indicating hepatomegaly (Figure 1C). Additionally, both CHD and IHD groups exhibited significantly elevated serum total cholesterol, LDL, and HDL (measured at weeks 4, 8, and 10), whereas no significant increase in triglycerides was observed (Figure 1D–G). Compared with the ND group, the CHD and IHD groups exhibited significantly higher levels of fasting blood glucose, serum insulin, and HOMA-IR (Supplementary Figure S1A–C). No differences in blood urea nitrogen or creatinine were observed among the three groups (Supplementary Figure S1D,E).

### 3.2. Impact of Dietary Patterns on Liver Function and Histomorphology

Hepatic function assessment showed that serum ALT was elevated only in the IHD group, whereas AST increased in both dietary groups (Figure 1H,I). Both diets raised IL-6 and TNF-α levels, but the TNF-α increase was significantly greater in the IHD group (Figure 1J,K). Gross liver examination revealed marked hepatomegaly with pale/yellowish discoloration and blunted edges in both CHD and IHD groups (Figure 1L). Histopathology confirmed severe hepatic steatosis. H&E and Oil Red O staining showed significant lipid accumulation in hepatocytes. Quantitative analysis indicated that both the number and total area of lipid droplets increased in CHD and IHD groups, with further elevation in the IHD group (Figure 1L,M). Masson’s trichrome staining revealed collagen deposition, and the fibrotic area was significantly increased in the IHD group (Figure 1L,N). Hepatic oxidative stress markers showed markedly elevated MDA, particularly in the IHD group, and decreased GSH levels in both groups (Figure 1O,P). Collectively, both continuous and intermittent HFHC feeding induced liver dysfunction and morphological changes. Critically, the intermittent HFHC diet caused more severe liver injury, as evidenced by exacerbated steatosis and significant fibrosis.

### 3.3. Hepatic Transcriptomic Changes in Response to Continuous vs. Intermittent HFHC Diet

**Figure 2:**
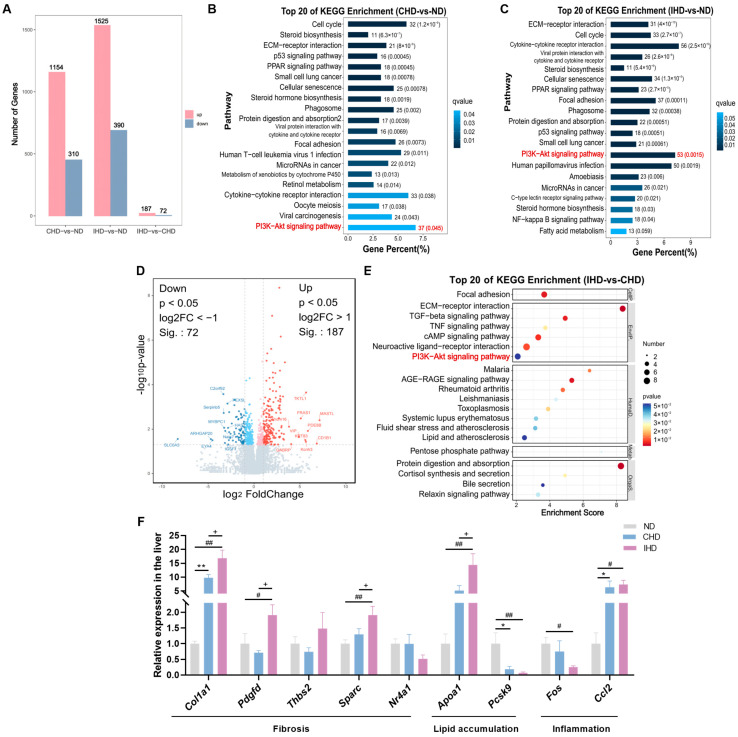
Transcriptomic profiling reveals differential gene expression in guinea pigs subjected to different dietary patterns. (**A**) Number of differentially expressed genes (DEGs) between groups; (**B**) KEGG pathway enrichment analysis of DEGs between the CHD and ND groups; (**C**) KEGG pathway enrichment analysis of DEGs between the IHD and ND groups; (**D**) volcano plot of DEGs comparing the IHD and CHD groups; (**E**) KEGG pathway enrichment analysis of DEGs between the IHD and CHD groups; (**F**) qRT-PCR validation of selected DEGs. Data are presented as mean ± SEM (*n* = 6 per group). One-way ANOVA with a multiple comparison test was used for statistical analysis. * *p* < 0.05, ** *p* < 0.01 (CHD vs. ND); # *p* < 0.05, ## *p* < 0.01 (IHD vs. ND); + *p* < 0.05 (IHD vs. CHD).

Transcriptomic sequencing of liver samples from ND, CHD, and IHD groups revealed substantial gene expression changes. Compared to ND, CHD showed 1154 upregulated and 310 downregulated genes; IHD showed 1525 upregulated and 390 downregulated. Direct comparison (IHD vs. CHD) identified 187 upregulated and 72 downregulated genes (Figure 2A). KEGG enrichment analysis showed the PI3K-Akt signaling pathway as the most significantly enriched in CHD vs. ND (37 genes), IHD vs. ND (53 genes), and crucially, also in the direct IHD vs. CHD comparison (Figure 2B–E). Given the established role of PI3K-Akt in MASLD, inflammation, and fibrosis [22,23], we validated nine candidate genes by qRT-PCR, grouped into three PI3K-Akt-regulated axes. First, fibrosis-related genes (*Col1a1*, *Pdgfd*, *Sparc*) were significantly upregulated in IHD versus ND and CHD, mirroring the aggravated fibrosis phenotype. *Col1a1* encodes the principal fibrillar collagen, *Pdgfd* drives hepatic stellate cell proliferation, and *Sparc* facilitates collagen assembly; all are downstream transcriptional targets of PI3K-Akt. Second, among lipid metabolism genes, *Apoa1* was selectively elevated in IHD, likely as a compensatory response to cholesterol overload, whereas *Pcsk9* was suppressed in both HFHC groups. Given that Akt signaling transcriptionally inhibits *Pcsk9*, this suppression would increase hepatic LDLR levels, potentially aggravating cholesterol uptake and lipotoxicity. Third, the pro-inflammatory chemokine *Ccl2*, a key driver of macrophage recruitment, was upregulated in both HFHC groups (Figure 2F). Collectively, this coordinated dysregulation supports a model wherein IHD-induced PI3K-Akt activation simultaneously promotes fibrogenesis, perturbs cholesterol handling, and amplifies inflammatory signaling.

### 3.4. Serum Metabolomics Links Enterolactone Deficiency to Aggravated Liver Injury in IHD

**Figure 3:**
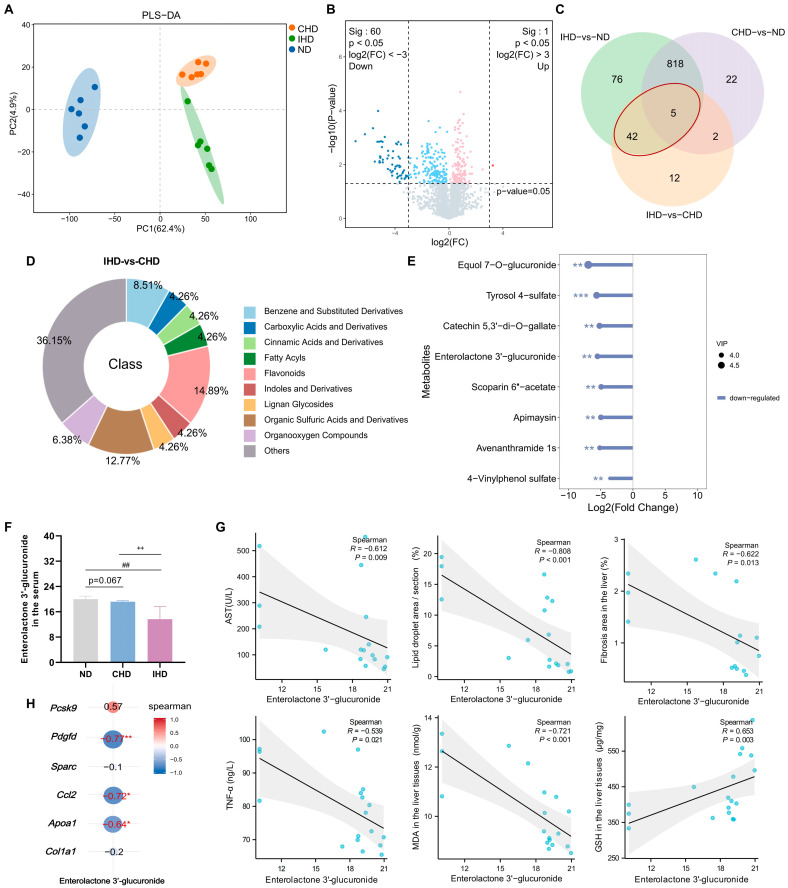
Comparison of the effects of continuous and intermittent HFHC diet on serum metabolites in guinea pigs. (**A**) PLS-DA score plot showing distinct separation of serum metabolite profiles among the ND, CHD, and IHD groups; (**B**) volcano plot of differential metabolites between the IHD and CHD groups; (**C**) Venn diagram illustrating the overlap of differential metabolites across the three groups; (**D**) classification of the 47 differential metabolites identified in the IHD group compared with the CHD and ND groups; (**E**) eight key differential metabolites between the IHD and CHD groups; (**F**) levels of enterolactone 3′-glucuronide in the three groups; (**G**) correlation analysis between enterolactone 3′-glucuronide levels and phenotypic parameters; (**H**) correlation analysis between enterolactone 3′-glucuronide levels and differentially expressed genes. *n* = 6 in each group. ## *p* < 0.01 (IHD vs. ND); ++ *p* < 0.01 (IHD vs. CHD). * *p* < 0.05, ** *p* < 0.01, and *** *p* < 0.001 in panels (**E**,**H**).

Untargeted metabolomics of serum from ND, CHD, and IHD guinea pigs showed distinct PLS-DA separation among groups, with CHD and IHD clustering closer together than to ND (Figure 3A). Compared to ND, CHD had 725 upregulated and 122 downregulated metabolites; IHD had 761 upregulated and 180 downregulated. Direct IHD vs. CHD comparison revealed only 1 upregulated and 60 downregulated metabolites (Figure 3B, Supplementary Figure S2A). Differentially abundant metabolites were mainly steroids/steroid derivatives, fatty acyls, and carboxylic acids/derivatives (Supplementary Figure S2B). The top 35 shared metabolites between CHD vs. ND and IHD vs. ND were predominantly glycerophosphocholines, fatty acyls, sphingolipids, and steroids/steroid derivatives (Supplementary Figure S2C). KEGG enrichment identified primary bile acid biosynthesis as the most significantly altered pathway in both diet groups vs. ND (Supplementary Figure S2D,E). Thus, both feeding patterns share a core metabolic disruption, with primary bile acid biosynthesis enrichment indicating reprogramming of hepatic cholesterol metabolism, and the predominance of lipid/sterol metabolites underscoring systemic lipid dyshomeostasis as a central event regardless of feeding pattern.

Venn diagram analysis identified shared and unique metabolite alterations among the three groups. Specifically, 823 metabolites were commonly altered in both CHD and IHD vs. ND (Figure 3C). Notably, 47 metabolites were commonly altered in both IHD vs. ND and IHD vs. CHD comparisons (Supplementary Table S2), among which flavonoids were the most enriched class (14.89%) (Figure 3D). Functional characterization of these 47 metabolites revealed 8 key metabolites linked to inflammation and oxidative stress: equol 7-O-glucuronide, apimaysin, tyrosol 4-sulfate, 4-vinylphenol sulfate, scoparin 6″-acetate, catechin 5,3′-di-O-gallate, enterolactone 3′-glucuronide, and avenanthramide 1s (Figure 3E). These are predominantly gut microbiota- or diet-derived phenolic compounds with anti-inflammatory and antioxidant properties. Integrative analysis identified enterolactone 3′-glucuronide as a key metabolite linking microbial dysbiosis to liver injury. Its circulating level negatively correlated with AST, TNF-α, MDA, lipid droplet area, and fibrosis area, and positively correlated with GSH (Figure 3F,G). Correlation analysis with six differentially expressed genes (*Col1a1*, *Pdgfd*, *Sparc*, *Apoa1*, *Pcsk9*, *Ccl2*) showed that *Pdgfd*, *Apoa1*, and *Ccl2* expression negatively correlated with enterolactone 3′-glucuronide levels (Figure 3H).

### 3.5. IHD-Associated Gut Microbiota Remodeling Mediates the Loss of Protective Enterolactone

**Figure 4:**
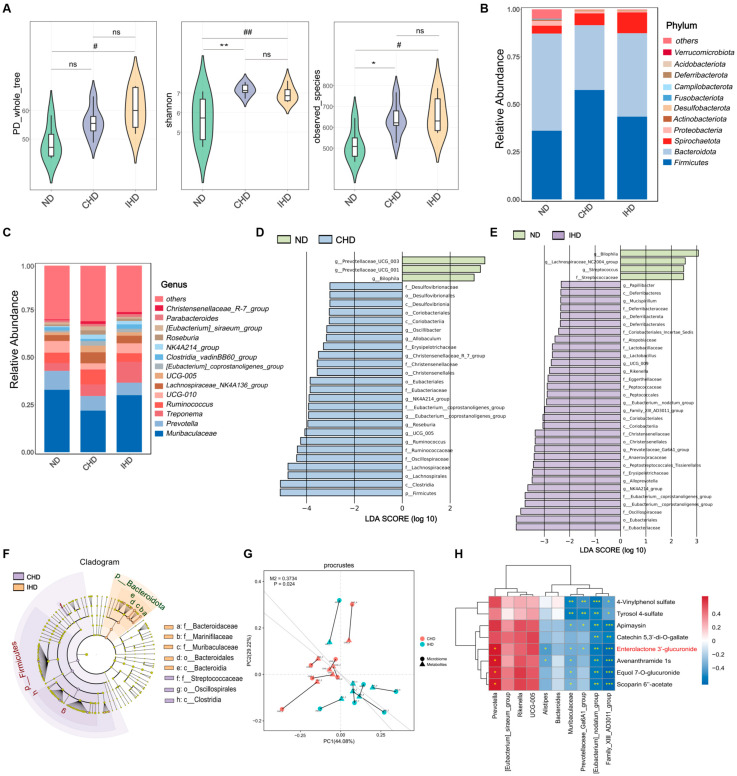
Gut microbiota sequencing revealed significant microbial changes induced by continuous and intermittent HFHC diet. (**A**) Comparison of α-diversity indices (PD_whole_tree and Shannon index) and observed species numbers among groups; (**B**,**C**) microbial composition comparison among groups at the phylum (**B**) and genus (**C**) levels; (**D**) differentially abundant microbes at various taxonomic levels between the CHD and ND groups. (**E**) Significantly different microbes at various taxonomic levels between the IHD and ND groups. (**F**) Cladogram illustrating differential microbes at various taxonomic levels between the IHD and CHD groups. (**G**) Procrustes analysis showing the overall association between gut microbiota profiles (sequencing data) and metabolomic profiles; (**H**) heatmap showing correlations between differentially abundant microbes and key differential metabolites in the IHD vs. CHD groups. *n* = 6 in each group. ns, not significant; * *p* < 0.05 and ** *p* < 0.01 (CHD vs. ND); # *p* < 0.05 and ## *p* < 0.01 (IHD vs. ND). * *p* < 0.05, ** *p* < 0.01, and *** *p* < 0.001 in panel (**H**).

Given that enterolactone 3′-glucuronide is a host-conjugated metabolite derived from the gut microbiota-produced enterolactone, we reasoned that gut microbial dysbiosis may act as an upstream driver of the metabolic dysregulation induced by continuous or intermittent HFHC feeding. To test this, we performed 16S rRNA sequencing on fecal samples from the ND, CHD, and IHD groups. Analysis revealed a significant increase in the number of observed species in both the CHD and IHD groups compared to the ND group. Furthermore, alpha-diversity indices (PD_whole_tree and Shannon index) were significantly elevated, with the IHD group showing the most pronounced increase (Figure 4A). Principal coordinate analysis (PCoA) of the microbial communities demonstrated a distinct separation among the ND, CHD, and IHD groups (Supplementary Figure S3A).

Next, differences in gut microbiota composition among the groups were compared. At the phylum level, the top three most abundant gut microbial taxa in guinea pigs were *Firmicutes*, *Bacteroidota*, and *Spirochaetota* (Figure 4B). At the genus level, the most abundant taxa were *Muribaculaceae*, *Prevotella*, and *Treponema* (Figure 4C). To further explore the differential gut microbiota, when comparing the CHD group to the ND group, the most significantly altered phyla were Firmicutes and *Deferribacterota* (Figure 4D, Supplementary Figure S3B). In contrast, the comparison between the IHD and ND groups revealed *Deferribacterota* as the most significantly changed phylum (Figure 4E, Supplementary Figure S3C). At the genus level, taxa that were significantly and commonly altered in both the CHD and IHD groups included *[Eubacterium]_coprostanoligenes_group*, *NK4A214_group*, and *Prevotellaceae _Ga6A1_group* (Supplementary Figure S3B,C). These results suggest that both the continuous and intermittent HFHC diets disrupted gut microbiota composition.

Direct comparison between the IHD and CHD groups by Linear Discriminant Analysis (LDA) identified *Firmicutes* and *Bacteroidota* as the most significantly altered phyla (Figure 4F), with *Muribaculaceae*, *Ruminococcus*, and *Bacteroides* representing the key discriminatory genera at the genus level (Supplementary Figure S3D). To determine whether these microbial shifts are linked to the metabolic disparities between the two groups, Procrustes analysis was performed and confirmed a significant association between gut microbiota composition and the metabolomic profiles (Figure 4G). Spearman correlation analysis between 10 differentially abundant genera and the 8 key metabolites revealed that the abundance of *Prevotella* was positively correlated with enterolactone 3′-glucuronide levels, whereas *Muribaculaceae*, *[Eubacterium]_nodatum_group*, *Family_XIII_AD3011_group*, *Alistipes* and *Prevotellaceae_Ga6A1_group* showed significant negative correlations (Figure 4H). These findings identify a distinct microbial signature that discriminates IHD from CHD and suggest that the selective depletion of *Prevotella*, coupled with the enrichment of other taxa, contributes to the reduced production of enterolactone, thereby providing a mechanistic link between dietary pattern-specific gut microbiota remodeling and aggravated liver injury.

### 3.6. Fecal Microbiota Transplantation Confirms That Gut Dysbiosis Exacerbates Liver Injury

**Figure 5:**
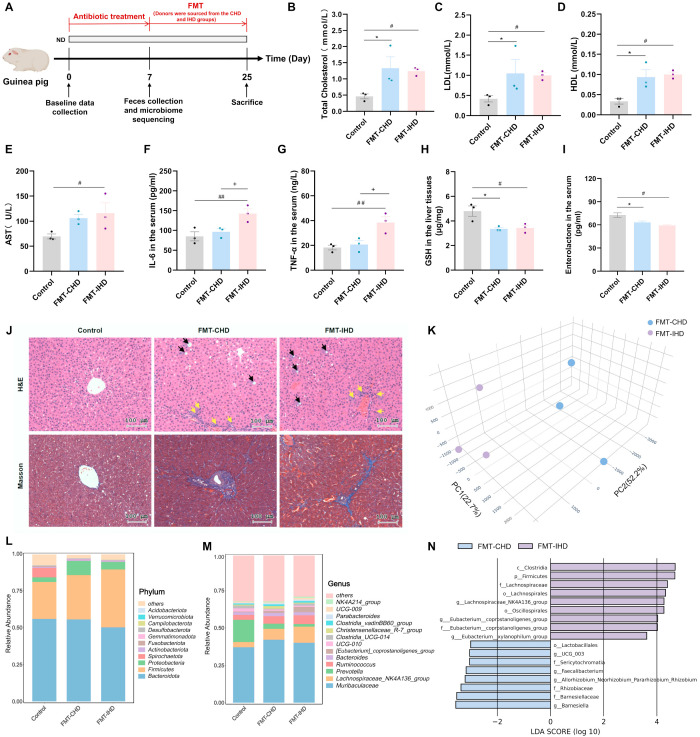
Fecal microbiota transplantation (FMT) from continuous and intermittent HFHC diet donors drives distinct phenotypes in recipient guinea pigs. (**A**) Schematic of the FMT experimental design; (**B**–**D**) serum levels of total cholesterol (**B**), LDL (**C**), and HD (**D**) in recipient guinea pigs; (**E**) Serum AST levels; (**F**,**G**) Serum IL-6 (**F**) and TNF-α (**G**) levels; (**H**) hepatic GSH levels; (**I**) serum enterolactone levels; (**J**) representative images of liver morphology (H&E and Masson’s trichrome staining), scale bar is 100 µm and the magnification is 200×, black arrows indicate lipid droplets and yellow arrows indicate inflammatory cell infiltrates; (**K**) principal component analysis (PCA) of gut microbiota composition among groups; (**L**,**M**) relative abundance of gut microbiota at the phylum (**L**) and genus (**M**) levels; (**N**) significantly different microbes at various taxonomic levels between the FMT-IHD and FMT-CHD groups. *n* = 3 in each group. Data are presented as mean ± SEM. One-way ANOVA with a multiple comparison test was used for statistical analysis. * *p* < 0.05 (FMT-CHD vs. Control); # *p* < 0.05, ## *p* < 0.01 (FMT-IHD vs. Control); + *p* < 0.05 (FMT-IHD vs. FMT-CHD).

Given the depletion of enterolactone and IHD-associated microbial shifts, we performed FMT to test whether the gut microbiota dysbiosis causally mediates aggravated liver injury (Figure 5A). Following antibiotic treatment in guinea pigs, fecal 16S rRNA sequencing showed significantly reduced observed species and alpha diversity (PD_whole_tree, Shannon) in the antibiotic-treated group vs. control (Supplementary Figure S4A). Microbial composition also markedly shifted, with Lactobacillus (~40%) and Klebsiella (~5–10%) becoming dominant while original genera diminished (Supplementary Figure S4B,C), confirming effective gut microbiota depletion.

Fecal microbiota from CHD or IHD donors was transplanted into antibiotic-treated recipients. Compared to controls, FMT recipients showed no significant differences in body weight, liver weight, or triglycerides (Supplementary Figure S4D–F), but total cholesterol, LDL, and HDL were significantly elevated in both FMT-CHD and FMT-IHD groups (Figure 5B–D). AST levels significantly increased only in the FMT-IHD group (Figure 5E). IL-6 and TNF-α were significantly higher in FMT-IHD vs. controls, and also significantly different between FMT-IHD and FMT-CHD (Figure 5F,G). Hepatic GSH was significantly reduced in both FMT groups, while MDA showed no differences (Figure 5H, Supplementary Figure S4G). Enterolactone levels were significantly reduced in both FMT groups (Figure 5I). Histology (H&E, Masson’s trichrome) revealed marked lipid droplets, inflammatory infiltration, and collagen deposition in FMT recipient livers (Figure 5J), recapitulating the aggravated liver injury seen in donors. 16S rRNA sequencing of FMT-recipient fecal samples showed distinct separation between FMT-CHD and FMT-IHD groups by PCoA (Figure 5K). Significant differences in microbial composition at phylum and genus levels were observed between FMT groups and controls (Figure 5L,M), and Figure 5N highlights differentially abundant taxa between FMT-CHD and FMT-IHD.

Collectively, these FMT experiments establish that gut microbiota dysbiosis induced by continuous or intermittent HFHC feeding is sufficient to transmit the pathogenic phenotype and corresponding hepatic steatosis, inflammation, and fibrosis in recipients, independent of direct dietary effects.

### 3.7. Dietary Lignan Restores Enterolactone and Alleviates IHD-Induced Liver Injury

Flaxseed is the richest dietary source of lignans, containing over 100 times the amount found in most other foods, with secoisolariciresinol diglucoside (SDG) as its predominant lignan [18,19]. Crucially, the anti-inflammatory and antioxidant benefits of flaxseed lignans are entirely dependent on their gut microbial conversion into the bioactive metabolite enterolactone [24,25,26,27,28,29]. We speculated that under IHD, reduced enterolactone bioavailability (reflected by decreased glucuronidated metabolite) attenuates hepatic protection and exacerbates injury. Thus, we hypothesized that dietary lignan supplementation could enhance enterolactone production, restore its bioavailability, and alleviate hepatic inflammation and injury.

**Figure 6:**
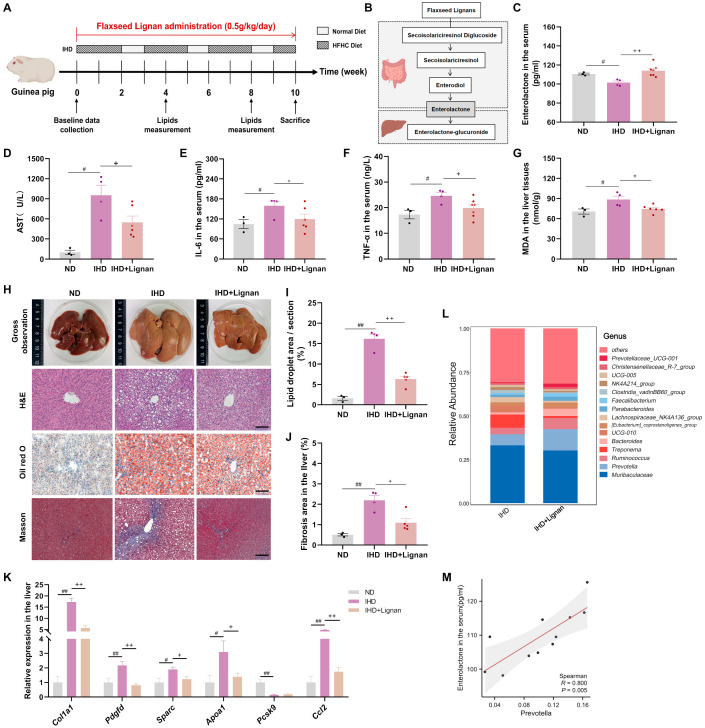
Intervention with flaxseed lignan extract alleviates the phenotypic alterations induced by an intermittent HFHC diet. (**A**) Schematic of the flaxseed lignan intervention experiment in guinea pigs. (**B**) Proposed metabolic pathway of flaxseed lignans in vivo. (**C**) Enterolactone level in the serum. (**D**) Serum AST levels. (**E**,**F**) Serum IL-6 (**E**) and TNF-α (**F**) levels. (**G**) Hepatic MDA levels. (**H**) Representative images of liver morphology (gross appearance, H&E staining, Oil Red O staining, and Masson staining), scale bar is 100 µm and the magnification is 200×. (**I**,**J**) Quantification of hepatic lipid droplet area (**I**) and fibrosis area (**J**). (**K**) qRT-PCR validation of key differentially expressed genes in liver tissue. (**L**) Comparative analysis of gut microbiota composition at the genus level between the IHD + Lignan and IHD groups. (**M**) Correlation between *Prevotella* abundance and enterolactone levels. *n* = 3 in ND group, *n* = 3–4 in IHD group and *n* = 6–7 in IHD + Lignan group. Data is presented as mean ± SEM. One-way ANOVA with a multiple comparison test was used for statistical analysis. # *p* < 0.05, ## *p* < 0.01 (IHD vs. ND); + *p* < 0.05, ++ *p* < 0.01 (IHD + Lignan vs. IHD).

To test this, we supplemented flaxseed lignans to IHD-fed guinea pigs (Figure 6A). The dominant lignan, secoisolariciresinol diglucoside (SDG), is metabolized by gut microbiota to enterolactone, which is then glucuronidated in the liver (Figure 6B). Our intervention successfully elevated circulating enterolactone levels (Figure 6C). Notably, IHD-induced changes in body weight, food intake, and liver weight were reverted to control levels (Supplementary Figure S5A–C). Lignan supplementation significantly alleviated liver injury (reduced serum AST, Figure 6D), suppressed hepatic inflammation (lower IL-6 and TNF-α, Figure 6E,F), and mitigated oxidative stress (reduced MDA, Figure 6G). Histopathology showed reduced lipid droplet accumulation and collagen deposition (Figure 6H–J). Dyslipidemia was not significantly altered (Supplementary Figure S5D–G). qRT-PCR confirmed that fibrosis-related (*Col1a1*, *Pdgfd*, *Sparc*), lipid metabolism-related (*Apoa1*), and pro-inflammatory (*Ccl2*) genes were significantly downregulated vs. IHD (Figure 6K).

Gut microbiota analysis of the lignan supplementation group showed significantly reduced observed species and alpha diversity (Chao1, PD_whole_tree, ACE) compared to untreated IHD (Supplementary Figure S5H). Microbial composition differences were observed between groups. At the genus level, abundances of *Prevotella*, *Treponema*, *Bacteroides*, and *NK4A214_group* were restored to normal (Figure 6L, Supplementary Figure S5I,J). *Prevotella* abundance positively correlated with enterolactone levels, suggesting it may be a key genus for enterolactone production from dietary lignans, with higher abundance promoting systemic bioavailability of this bioactive enterolignan (Figure 6M).

### 3.8. In Vitro Effects of Enterolactone on Palmitic Acid-Challenged HepG2 Cells

To further validate the direct effect of enterolactone on hepatic cells, we performed in vitro experiments using HepG2 cells. The CCK-8 assay showed that co-treatment with PA and 50 μM ENL significantly reduced cell proliferation and viability compared with the control; therefore, 10 μM and 30 μM ENL were selected for subsequent experiments (Supplementary Figure S6A). ROS measurement revealed that PA treatment markedly increased intracellular ROS levels, and this increase was significantly attenuated by co-treatment with 30 μM ENL (Supplementary Figure S6B). TG and TC quantification showed that PA significantly elevated both TG and TC contents, whereas co-treatment with 30 μM ENL significantly reduced both TG and TC, and 10 μM ENL significantly reduced TC content (Supplementary Figure S6C,D). Consistently, Oil Red O staining demonstrated that 30 μM ENL significantly decreased lipid accumulation compared with the PA-treated group (Supplementary Figure S6E). qRT-PCR analysis indicated that PA significantly upregulated the mRNA expression of *PDGFD*, *SPARC*, and *APOA1* relative to the control group. Both 10 μM and 30 μM ENL significantly downregulated *PDGFD* expression, and 30 μM ENL also significantly reduced *APOA1* expression compared with the PA group (Supplementary Figure S6F).

## 4. Discussion

**Figure 7:**
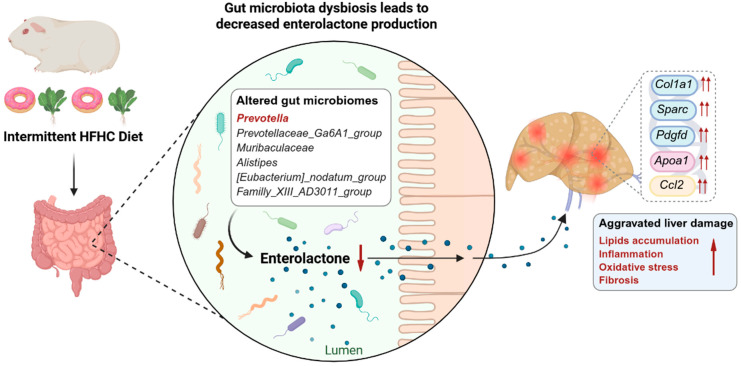
Intermittent high-fat high-cholesterol diet exacerbates liver injury via disruption of the gut microbiota–enterolactone axis. Schematic illustration of the proposed pathogenic mechanism. Unlike continuous high-cholesterol diet (CHD), the intermittent high-fat high-cholesterol diet (IHD) specifically reshapes the gut microbiota, leading to a marked reduction in the microbiota-derived protective metabolite enterolactone. This enterolactone deficiency drives aggravated hepatic steatosis, inflammation, oxidative stress, and fibrosis. Critically, dietary supplementation with flaxseed lignan restores enterolactone production by beneficially modulating the gut microbial composition, which in turn reverses these pathological features and attenuates liver injury. Created with BioRender.com.

This study presents the first integrated multi-omics comparison of serum metabolomic, gut microbiota, and hepatic transcriptomic profiles in guinea pigs exposed to continuous versus intermittent HFHC diets. While both dietary regimens induced hallmark MASLD features—including elevated serum lipids, hepatic steatosis, collagen deposition, and altered bile acid metabolism—the intermittent regimen provoked significantly more severe liver injury. Mechanistically, we attribute this exacerbated phenotype to a disruption of the gut microbiota–enterolactone axis. Intermittent HFHC feeding reshaped the gut microbiota in a manner that markedly depleted enterolactone, a microbiota-derived metabolite with well-documented anti-inflammatory and antioxidant properties. Fecal microbiota transplantation experiments confirmed the causal role of dysbiotic microbiota, as recipient animals recapitulated both the metabolic and hepatic injury phenotypes. Importantly, dietary supplementation with flaxseed lignan restored enterolactone production by modulating the gut microbiota and significantly attenuated hepatic inflammation, oxidative stress, and fibrosis specifically in the intermittent diet group (Figure 7).

The guinea pig is a highly suitable model for dietary studies due to its translational relevance in lipid metabolism [8,30]. Guinea pigs share key human metabolic features, including an LDL-dominant lipoprotein profile, hepatic cholesterol ester accumulation, and robust pathological responses-hallmarks of human MASLD [9,31]. This study validates these advantages. In the present study, HFHC-fed guinea pigs exhibited reduced body weight, a departure from classic obesity models that are primarily driven by their herbivorous physiology and the transient hypophagia that occurs when shifting from a high-fiber chow to a cholesterol-rich, energy-dense diet. A similar weight phenotype was reported by Ipsen et al. [32], and the absence of cachexia together with typical steatohepatitis on histology confirms that this weight change does not compromise the model. Guinea pigs also showed pronounced sensitivity to the HFHC diet, with significant elevations in total cholesterol and LDL emerging within just one week. The lack of a significant increase in triglycerides reflects their LDL-dominant lipoprotein metabolism and limited hepatic VLDL-TG secretion under cholesterol loading, a response consistently observed in previous guinea pig studies [32,33]. In contrast, commonly used mouse models have a plasma lipid profile substantially different from humans (high HDL, low LDL) due to natural CETP deficiency [34,35]. Establishing diet-induced MASLD/NASH with steatosis and metabolic disturbances in mice typically requires 12–16 weeks, and even longer for significant fibrosis [36,37,38]. In our study, continuous HFHC feeding for 7 weeks was sufficient to induce marked liver injury and characteristic hepatic features of MASLD in guinea pigs, substantially shortening the time needed to establish a robust MASLD model. Notably, intermittent HFHC induced more severe liver injury than continuous feeding, a finding relevant to human cyclical unhealthy eating patterns. A significant divergence in gut microbiome composition was observed between the two dietary groups. The exacerbated injury under intermittent feeding is primarily attributed to repeated metabolic and microbial stress from cyclical dietary shifts, which disrupt microbial community stability, select dysbiotic microbiota, and compromise intestinal barrier function [39,40]. This dysbiosis drives reduced circulating protective metabolites (e.g., enterolactone) and promotes hepatic inflammation and oxidative stress, collectively accelerating liver damage.

Moreover, our findings provide direct evidence that dietary flaxseed lignan supplementation effectively rescues the exacerbated liver injury triggered by an intermittent HFHC diet. This therapeutic effect is mechanistically rooted in the restoration of the disrupted gut microbiota–enterolactone axis. Our untargeted metabolomics identified enterolactone 3′-glucuronide, a major circulating glucuronide conjugate of the lignan metabolite enterolactone, as the most prominently depleted protective metabolite in the IHD group. Its circulating levels showed robust negative correlations with AST, TNF-α, MDA, hepatic lipid droplet area, and fibrosis area, and a positive correlation with GSH. By supplying its dietary precursor (flaxseed lignan, which is converted to enterolactone by the gut microbiota), flaxseed lignan supplementation directly counteracted this deficit, significantly elevating systemic enterolactone levels and concomitantly attenuating hepatic inflammation, oxidative stress, and fibrosis [41,42,43]. These observations establish enterolactone as a functional mediator of the axis, whose loss is both a biomarker and a mechanistic driver of IHD-specific liver pathology, rather than a mere bystander.

Critically, the efficacy of flaxseed lignan supplementation depends on the presence of a competent gut microbiota capable of converting lignans into enterolactone [44,45,46,47]. This conversion is a well-characterized, multi-step process orchestrated by distinct bacterial consortia. Plant lignans such as secoisolariciresinol diglucoside (SDG) are initially deglycosylated by bacterial β-glucosidases (e.g., from *Bacteroides* species) to release the aglycone secoisolariciresinol (SECO). Subsequent demethylation and dehydroxylation, carried out primarily by *Eggerthella lenta* along with certain *Clostridium* species, convert SECO to the intermediate enterodiol. A final dehydrogenation step, performed by bacteria such as *Lactonifactor longoviformis*, yields enterolactone [48,49]. Notably, PCR-DGGE and in vitro pure culture studies have shown that saccharolytic genera such as *Prevotella* harbor an extensive capability to deglycosylate plant lignans, and that *Prevotella* spp. are the main converters of SDG into SECO in the rumen [50]. This provides a mechanistic underpinning for the tight positive correlation between Prevotella and enterolactone observed in our study and suggests that the IHD-induced loss of *Prevotella* may directly impair the initial deglycosylation step, thereby blunting endogenous enterolactone production. Our 16S rRNA sequencing revealed that IHD-induced dysbiosis is characterized by a marked depletion of *Prevotella*, a genus positively correlated with enterolactone levels, alongside the expansion of potentially pro-inflammatory taxa such as *Alistipes*, *Muribaculaceae*, and the *[Eubacterium]_nodatum_group*. This concept is reinforced by our FMT experiments, which demonstrated that transfer of the dysbiotic IHD microbiota is sufficient to transmit the aggravated liver phenotype. By modulating this dysbiotic configuration, lignan supplementation likely achieved a dual restoration: it promoted the re-emergence of lignan-metabolizing bacteria, thereby reinstating endogenous enterolactone production, while concurrently suppressing pathobionts that contribute to endotoxemia and hepatic inflammation. Thus, flaxseed lignan intervention functions not as a simple metabolite replacement therapy, but as a microbiota-targeted prebiotic strategy that reconfigures a pathologic microbial ecosystem toward a health-promoting state, restoring the gut microbiota–enterolactone axis as a central protective mechanism against diet-pattern-driven MASLD.

The role of the PI3K-Akt pathway in MASLD is well documented [51]. In this study, hepatic transcriptomic analysis identified the PI3K-Akt signaling pathway as the most significantly enriched pathway in the IHD group. Although direct phospho-protein measurements were not performed, the concerted transcriptional changes in five qPCR-validated downstream targets provide functional signatures indicative of PI3K-Akt pathway activation. Specifically, *Pcsk9* was suppressed in both HFHC groups. Given that PI3K-Akt signaling transcriptionally represses *Pcsk9*, its downregulation implies increased hepatic LDLR levels, which could aggravate cholesterol uptake and contribute to lipotoxicity; this is further supported by the concomitant elevation of *Apoa1* in IHD, reflecting perturbed lipid handling. The pro-inflammatory chemokine *Ccl2* was upregulated in both HFHC groups, consistent with sustained hepatic inflammation. Fibrosis-related genes (*Col1a1*, *Pdgfd*, *Sparc*) were selectively upregulated in IHD, delineating a fibrogenic transcriptional program. These transcriptional signatures suggest that PI3K-Akt activation likely engages multiple hepatic cell types: hepatocytes as the primary drivers of steatosis and inflammatory signaling, hepatic stellate cells as the source of the fibrogenic program, and macrophages as potential amplifiers of injury through *Ccl2*-mediated recruitment. Notably, flaxseed lignan supplementation largely restored the expression of these downstream targets, indicating that dietary lignans counteract the transcriptional consequences of PI3K-Akt overactivation, potentially through a coordinated protective effect across these cell populations. Intermittent HFHC feeding depletes enterolactone, which likely relieves its tonic inhibition on the pathway, thereby triggering steatotic, inflammatory, and fibrotic transcriptional programs driven, at least in part, by PI3K-Akt activation. Restoration of enterolactone via lignan supplementation re-establishes this inhibition and alleviates liver injury. This model is consistent with reports that lignan metabolites can modulate PI3K-Akt activity in other tissues [52,53], and extends these observations to the gut–liver axis for the first time. However, direct kinetic evidence that enterolactone inhibits hepatic Akt phosphorylation remains to be established; future studies using phospho-specific protein analysis will be required to validate this proposed mechanism.

Our study carries direct implications for clinical nutritional intervention by demonstrating that cyclical dietary exposure (weekly alternation between an HFHC diet and normal chow) per se provokes more severe liver injury, revealing a novel pathogenic factor independent of total caloric intake. This underscores a critical yet underappreciated concept: the deleterious impact of an unhealthy diet is determined not only by “what” and “how much” is consumed, but also by the stability of the dietary pattern over time-the rhythm of alternating between healthy and unhealthy eating. Our results demonstrate that cyclical dietary exposure can promote MASLD and highlight a potential avenue for targeted dietary strategies.

This study has several limitations. First, the intermittent feeding protocol (weekly alternation between HFHC and normal diets) does not directly model irregular meal timing patterns; any extrapolation to such patterns or populations should therefore be regarded as a possible implication rather than a direct experimental conclusion. Second, all multi-omics assessments were performed only at the study endpoint, precluding the capture of dynamic microbial and metabolic changes expected from the cyclical dietary regimen and limiting conclusions to endpoint associations. Third, dietary lignan supplementation was provided using a commercial flaxseed lignan extract containing 40% SDG, not as purified enterolactone; consequently, the observed effects cannot be attributed exclusively to enterolactone and may involve other lignan metabolites or matrix-associated components. Fourth, the identification of enterolactone and related metabolites relied partly on untargeted metabolite annotations (e.g., accurate mass and spectral library matching) without confirmation by authentic standards, which introduces a degree of annotation uncertainty. Fifth, enterolactone was quantified only in peripheral serum, and local intestinal levels (e.g., in cecal contents or feces) were not measured. This limits our ability to directly demonstrate that microbial lignan conversion in the gut is proportionally restored in tandem with key taxa such as *Prevotella*, and to fully close the causal loop of the gut–liver axis. Finally, PI3K-Akt pathway activation was inferred from transcriptomic pathway enrichment and qPCR-validated downstream gene expression; direct measurement of Akt phosphorylation or other biochemical indicators of pathway activity was not feasible owing to the lack of commercially available antibodies that reliably cross-react with guinea pig proteins, a limitation that is especially pronounced for phospho-specific reagents.

## 5. Conclusions

This multi-omics study demonstrates that an intermittent HFHC diet induces more severe hepatic steatosis, inflammation, and fibrosis than a continuous regimen in guinea pigs, a model that recapitulates aspects of human lipid metabolism. Mechanistically, the aggravated liver injury was associated with gut microbiota dysbiosis and a marked reduction in the microbial metabolite enterolactone. Dietary flaxseed lignan supplementation increased enterolactone levels, partially restored microbial homeostasis, and modulated the expression of PI3K-Akt downstream targets, thereby alleviating liver injury. These findings suggest that alterations in the gut microbiota–enterolactone axis may contribute to diet-periodicity-driven liver injury and highlight its potential involvement in disease progression. Modulation of this axis through dietary intervention with flaxseed lignans may represent a promising nutritional strategy for managing MASLD associated with cyclical dietary exposure.

## Data Availability

The datasets generated and/or analyzed during the current study are available in the Genome Sequence Archive (GSA) at the National Genomics Data Center, China National Center for Bioinformation/Beijing Institute of Genomics, Chinese Academy of Sciences, under Bioproject accession number PRJCA056715. The dataset accession numbers are CRA037988 and CRA037844, and the data is publicly accessible at https://ngdc.cncb.ac.cn/gsa (accessed on 1 February 2026). The metabolomic data have been deposited in the OMIX database under the dataset identifier OMIX014597.

## Supplementary Materials

The following supporting information can be downloaded at https://www.mdpi.com/article/10.3390/nu18152573/s1, Table S1. Real-time PCR primers; Table S2. Key differential metabolites altered in guinea pigs on an intermittent HFHC diet compared to those on a continuous HFHC diet and a normal diet; Figure S1. Comparison of the effects of continuous and intermittent HFHC diet on physiological characteristics in guinea pigs; Figure S2. Comparison of serum metabolic profiles in guinea pigs under continuous and intermittent HFHC diets; Figure S3. Differential alterations in the gut microbiome across dietary patterns; Figure S4. Efficiency of gut microbiome elimination following antibiotic treatment in guinea pigs and physiological characteristics after fecal microbiota transplantation from donor animals fed continuous or intermittent HFHC diets; Figure S5. Effects of flaxseed lignan treatment on physiological characteristics and gut microbiota in guinea pigs; Figure S6. In vitro effects of enterolactone on palmitic acid-challenged HepG2 cells.

## Abbreviations

MASLD	Metabolic dysfunction-associated steatotic liver disease
SDG	Secoisolariciresinol diglucoside
HFHC	High-fat, high-cholesterol
ND	Normal diet
CHD	Continuous high-fat, high-cholesterol diet
IHD	Intermittent high-fat, high-cholesterol diet
TG	Triglycerides
TC	Total cholesterol
LDL	Low-density lipoprotein
HDL	High-density lipoprotein
VLDL	Very low-density lipoprotein
ALT	Alanine aminotransferase
AST	Aspartate aminotransferase
SOD	Superoxide dismutase
MDA	Malondialdehyde
GSH	Glutathione
PLS-DA	Partial least squares-discriminant analysis
CETP	Cholesteryl ester transfer protein
Abx	Antibiotic treatment
FMT	Fecal microbiota transplantation
