# Supplementary material for: Dietary Periodicity Disrupts the Gut Microbiota–Enterolactone Axis to Exacerbate MASLD in a Translational Guinea Pig Model
Source: Nutrients. 2026 Aug 6;18(15):2573. doi: 10.3390/nu18152573 (PMC13467970; doi:10.3390/nu18152573)
Supplement: Supplementary file 1 [file nutrients-18-02573-s001.zip › nutrients-4435388-supplementary.pdf]

Supplementary Table S1. Real-time PCR primers

Gene	Full Name	Forward Primer	Reverse Primer
Col1a1	***Collagen Type I Alpha 1 Chain***	ATGATGCCAATGTGGTCCGT	TCGATCCAGTACTCTCCGCT
Apoa1	***Apolipoprotein A1***	GGGCCAACAGCTAAACCTGA	GGTCCTCGCTCACTATCTGC
Pdgfd	***Platelet-Derived Growth Factor D***	ATCTTAGGCGCGATGAGAGC	TCCTGGGGTAGTTGTTCGGA
Nr4a1	***Nuclear Receptor Subfamily 4 Group A Member 1***	CTGCAAAAGTTGGGGGAGTG	CTTGGTGCTGGTGTCCCATA
Ccl2	***C-C Motif Chemokine Ligand 2 (MCP-1)***	GCTGGTCATAGAAGCCACCT	CCCTCTTCAGTGGGATCTGTT
Thbs2	***Thrombospondin 2***	TTTTATATACAGGTGGCGCAGC	CACCGATGGTCTTCCGGTTTA
Pcsk9	***Proprotein Convertase Subtilisin/Kexin Type 9***	AAAGGAGTACAGTGCCCCCA	ACACTTGCTTGCCTGTCTGT
Fos	***FBJ murine osteosarcoma viral oncogene homolog***	CCTCGCTCAGCTTTCACTCA	GATCCGTACAGAAGTCCTGCG
Sparc	***Secreted Protein Acidic and Cysteine Rich***	GCGAGTTTGAGAAGGTGTGC	TTTGCAAGGCCCGATGTAGT
Gapdh	***Glyceraldehyde-3-phosphate dehydrogenase***	TCGGAGTGAACGGATTTGGC	GGAACTTGCCGTGGGTAGAA
PDGFD (human)	***Platelet-Derived Growth Factor D***	CGCTGTCTCTAGTCGCTGAT	AGGTGATTGCTCTCATCTCGC
SPARC (human)	***Secreted Protein Acidic and Cysteine Rich***	GCCTGATGAGACAGAGGTGG	GTTCTGGCAGGGATTTTCCG
APOA1 (human)	***Apolipoprotein A1***	AGAGACTGCGAGAAGGAGGTC	GGGACACATAGTCTCTGCCG
GAPDH (human)	***Glyceraldehyde-3-phosphate dehydrogenase***	TCGGAGTCAACGGATTTGGT	TTCCCGTTCTCAGCCTTGAC

Supplementary Table S2. Key differential metabolites altered in guinea pigs on an intermittent HFHC diet compared to those on a continuous HFHC diet and a normal diet

No.	Putatively annotated metabolites	ND （N=6)	CHD（N=6)	IHD（N=6)	VIP (CHD-vs-ND)	VIP (IHD-vs-CHD)	VIP (IHD-vs-ND)
1	**Equol 7-O-glucuronide**	20.42±2.26	18.5±1.28	11.58±3.65##++	0.64	4.93	1.74
2	**Sulfometuron-methyl**	18.86±1.04	17.61±0.62	11.37±3.14##++	0.59	4.74	1.62
3	**Tyrosol 4-sulfate**	19.77±1.83	17.74±0.38	12.11±2.66##++	0.76	4.5	1.65
4	**Enterolactone 3'-glucuronide**	20.02±0.95	19.17±0.34	13.67±4.04##++	0.45	4.15	1.4
5	**Phe-Asn-Tyr**	16.32±3.18	17.1±0.74	11.80±2.89#++	0.25	4.31	1.07
6	**2-(1,3-Dioxo-1,3-dihydro-isoindol-2-yl)-3-(1H-indol-3-yl)-propionic acid**	19.72±1.35	16±1.3*	10.75±1.62##++	1.17	4.49	1.86
7	**Catechin 5,3'-di-O-gallate**	17.57±2.11	16.35±1.17	11.17±2.66##++	0.44	4.28	1.46
8	**Ceroptene**	20.46±0.94	19.51±0.31	14.35±3.84##++	0.5	4.03	1.37
9	**Avenanthramide 1s**	20.34±0.87	19.38±0.53	14.23±3.74##++	0.5	3.97	1.38
10	**2,8-Dihydroxyquinoline-beta-D-glucuronide**	17.9±4.09	17.34±1.07	12.23±3.32#++	0.15	4.1	1.2
11	**Afzelechin 3-O-alpha-L-rhamnopyranoside**	17.5±3.77	17.4±0.42	12.32±3.45#++	0.02	4.08	1.12

12	**1-(3,5-Dichlorophenyl)pyrrole-2,3,5-triol**	17.12±1.7	16.66±1.03	11.63±2.61$^{\#\#++}$	0.2	4.21	1.34
13	**Apimaysin**	18±1.01	16.93±0.32	11.96±2.97$^{\#\#++}$	0.54	4.13	1.42
14	**Scoparin 6''-acetate**	17.88±2.62	16.02±1.27	11.09±2.46$^{\#\#++}$	0.6	4.14	1.5
15	**Enterolactone 3''-glucuronide**	21.59±0.88	20.64±0.29	15.73±3.65$^{\#\#++}$	0.51	3.84	1.35
16	**Quinaldine**	19.44±0.81	18.02±0.8	13.12±3.42$^{\#\#++}$	0.66	3.92	1.44
17	**Methylpyrogallol sulfate 1**	16.63±4.1	16.24±1.25	11.48±2.19$^{\#++}$	0.12	3.95	1.17
18	**alpha-N-Dichloroacetyl-p-aminophenylserinol**	21.76±1.78	19.04±0.67	14.47±3.15$^{\#\#++}$	0.94	3.92	1.58
19	**(2S)-4,5,7-Trihydroxy-2-(3,4,5-trihydroxyphenyl)-4H-chromen-3-one**	18±4.09	16.81±1.61	12.28±3.40$^{\#++}$	0.32	3.69	1.2
20	**glas#1**	18.97±1.6	19.12±0.5	14.60±2.81$^{\#\#++}$	0.07	3.84	1.12
21	**Anileridine**	15.97±2.98	15.99±2.02	11.50±2.25$^{\#++}$	0	3.72	1.1
22	**1alpha,25-dihydroxy-11alpha-[(1S)-oxiranyl]vitamin D3**	18.33±2.13	18.9±1.29	14.54±3.47$^{\#+}$	0.21	3.4	0.94
23	**5-Hydroxy-6-methoxyindole glucuronide**	18.86±1.89	14.35±3.75*	10.09±0$^{\#\#+}$	1.11	3.54	1.85
24	**N-Acetyl-L-glutamyl 5-phosphate**	20.63±0.66	19.16±0.64	14.94±3.88$^{\#\#+}$	0.71	3.45	1.31
25	**Delapril diacid**	17.51±4.1	16.08±1.52	11.87±2.87$^{\#++}$	0.39	3.55	1.22

26	**Thiacloprid**	18.18±0.62	16.65±1.06	12.47±2.72##++	0.68	3.65	1.4
27	**2-Methoxyidazoxan**	18.01±1.04	17.33±0.86	13.19±2.49##++	0.33	3.57	1.26
28	**Resorufin**	16.13±1.9	15.91±1.7	11.91±2.84#+	0.08	3.46	1.09
29	**Catechin 7,3'-di-O-gallate**	18.22±1.87	17.68±0.69	13.72±3.08#+	0.22	3.38	1.12
30	**Mukonal**	17.58±1.5	15.23±1.18	11.31±1.92##++	0.85	3.69	1.5
31	**O-Ethyl s-(2-furylmethyl)thiocarbonate**	22.38±1.31	21.95±0.75	18.10±2.11##++	0.21	3.64	1.18
32	**XIPAMIDE**	18.3±1.94	15±2.67*	11.16±2.64##+	0.94	3.28	1.58
33	**2,6,3'-Trihydroxy-4'-methoxy-2-benzylco umaranone**	18.22±1.49	15.94±1.58	12.16±3.24##+	0.79	3.11	1.39
34	**4-Vinylphenol sulfate**	18.25±3.15	15±1.07*	11.25±1.82##++	0.93	3.59	1.54
35	**Harmalol**	16.7±3.29	15.3±0.98	11.56±2.37#++	0.42	3.44	1.2
36	**Sudoxicam**	19.99±0.56	18.44±0.99	14.77±2.78##+	0.7	3.3	1.31
37	**Aflatoxin B1 dialcohol**	19.23±2.09	19±0.58	15.36±3.37#+	0.09	3.05	0.97
38	**[4-[(E)-3-hydroxyprop-1-enyl]phenyl] hydrogen sulate**	20.05±1.17	18.94±0.9	15.30±2.40##++	0.5	3.42	1.25
39	**3-Hydroxyhippuric acid**	20.19±2.6	17.86±0.77	14.25±2.78##+	0.75	3.24	1.37
40	**2-(4-sulooxyphenyl)propanoic acid**	18.63±2.41	18.01±0.62	14.44±1.97##++	0.24	3.51	1.11

41	**Tetranor-5-NO2-CLA**	18.44±1.7	17.9±1.33	14.46±2.78$^{\#+}$	0.21	3.06	1.05
42	**Eriodictyol 5-O-rhamnoside**	18.5±2.21	14.27±2.31*	10.86±1.89$^{\#\#+}$	1.15	3.14	1.67
43	**Cinnamyl alcohol**	16.3±2.05	16.13±1.41	12.91±2.78$^{\#+}$	0.06	2.97	0.91
44	**3- Acetylphenol sulfate**	18.95±1.8	16.8±0.59	13.62±2.91$^{\#\#+}$	0.79	3.07	1.29
45	**SCHEMBL2963001**	16.05±3.38	14.29±2.31	11.22±1.75$^{\#+}$	0.49	2.89	1.16
46	**O-methoxycatechol-O-sulphate**	23.06±2.32	20.16±0.22	17.13±1.71$^{\#\#++}$	0.94	3.19	1.43
47	**2-Amino-7-[(2R)-2,3-dihydroxypropanoyl]-5,6,7,8-tetrahydro-3H-pteridin-4-one**	20.48±1.04	19.32±0.34	16.32±2.06$^{\#\#++}$	0.57	3.04	1.17

Data are presented as mean±SEM (n=6) and represent total ion current (TIC)-normalized peak areas (arbitrary units). All metabolites listed are putatively annotated at Metabolomics Standards Initiative (MSI) Level 2 (based on accurate mass and MS/MS database matching) and have not been confirmed by authentic standards. A VIP value > 1.0 is generally considered indicative of a significant discriminant. *: $p < 0.05$, CHD vs ND; #: $p < 0.05$, IHD vs ND; ##: $p < 0.01$, IHD vs ND; +: $p < 0.05$, IHD vs CHD; ++: $p < 0.01$, IHD vs CHD.

ND: normal diet; CHD: continuous high-fat, high-cholesterol diet; IHD: intermittent high-fat, high-cholesterol diet; VIP: Variable Importance in Projection.

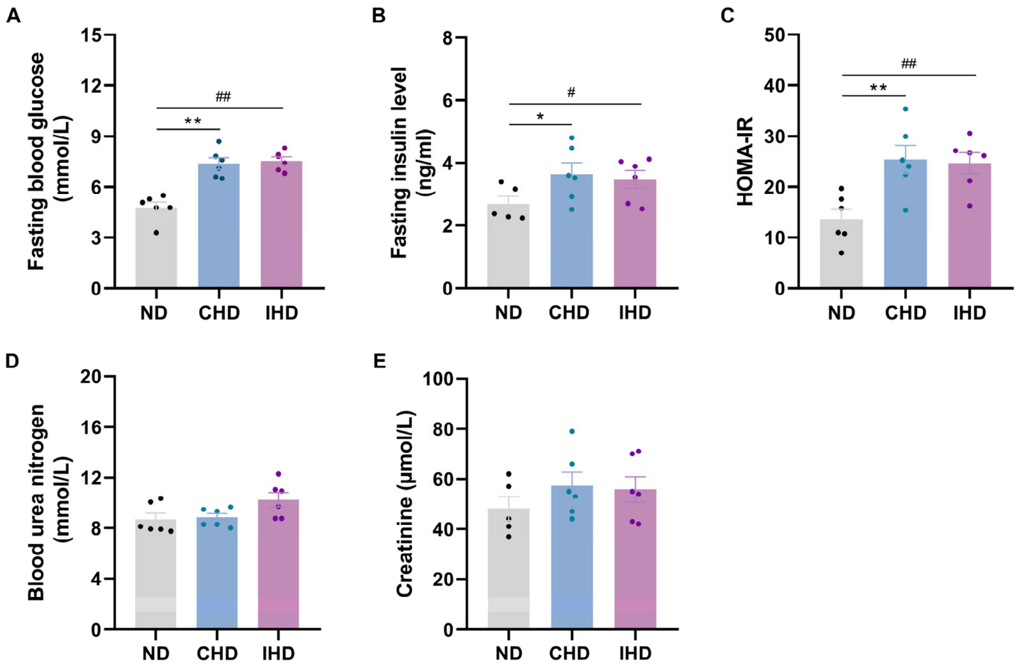

Supplementary Figure S1. Comparison of the effects of continuous and intermittent HFHC diet on physiological characteristics in guinea pigs.

A: Fasting blood glucose level; B: Fasting serum insulin level; C: HOMA-IR; D: Blood urea nitrogen (BUN) level; E: Serum creatinine level. Data are presented as mean ± SEM. N = 5-6 in each group. *: $p < 0.05$, CHD vs ND; **: $p < 0.01$, CHD vs ND; #: $p < 0.05$, IHD vs ND; ##: $p < 0.01$, IHD vs ND.

HFHC: high-fat, high-cholesterol; ND: normal diet; CHD: continuous high-fat, high-cholesterol diet; IHD: intermittent high-fat, high-cholesterol diet; HOMA-IR: homeostatic model assessment for insulin resistance.

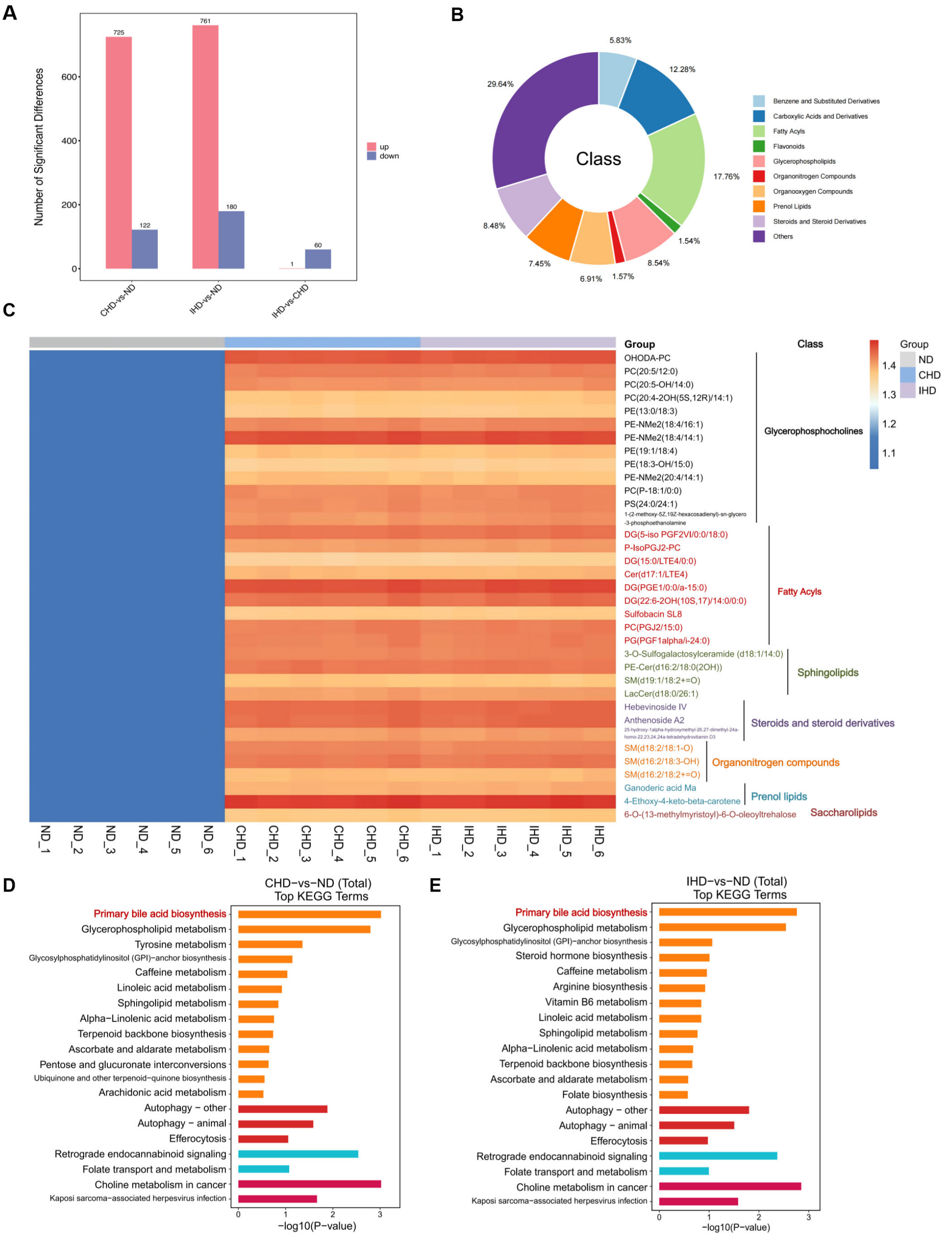

Supplementary Figure S2. Comparison of serum metabolic profiles in guinea pigs under continuous and intermittent HFHC diets.

A: Number of differential serum metabolites among groups; B: Classification of differential metabolites; C: Heatmap of metabolites commonly altered in both the CHD and IHD groups compared to the ND group; D: KEGG analysis of differential metabolites between the CHD and ND groups; E: KEGG analysis of differential metabolites between the IHD and ND groups.

HFHC: high-fat, high-cholesterol; ND: normal diet; CHD: continuous high-fat, high-cholesterol diet; IHD: intermittent high-fat, high-cholesterol diet.

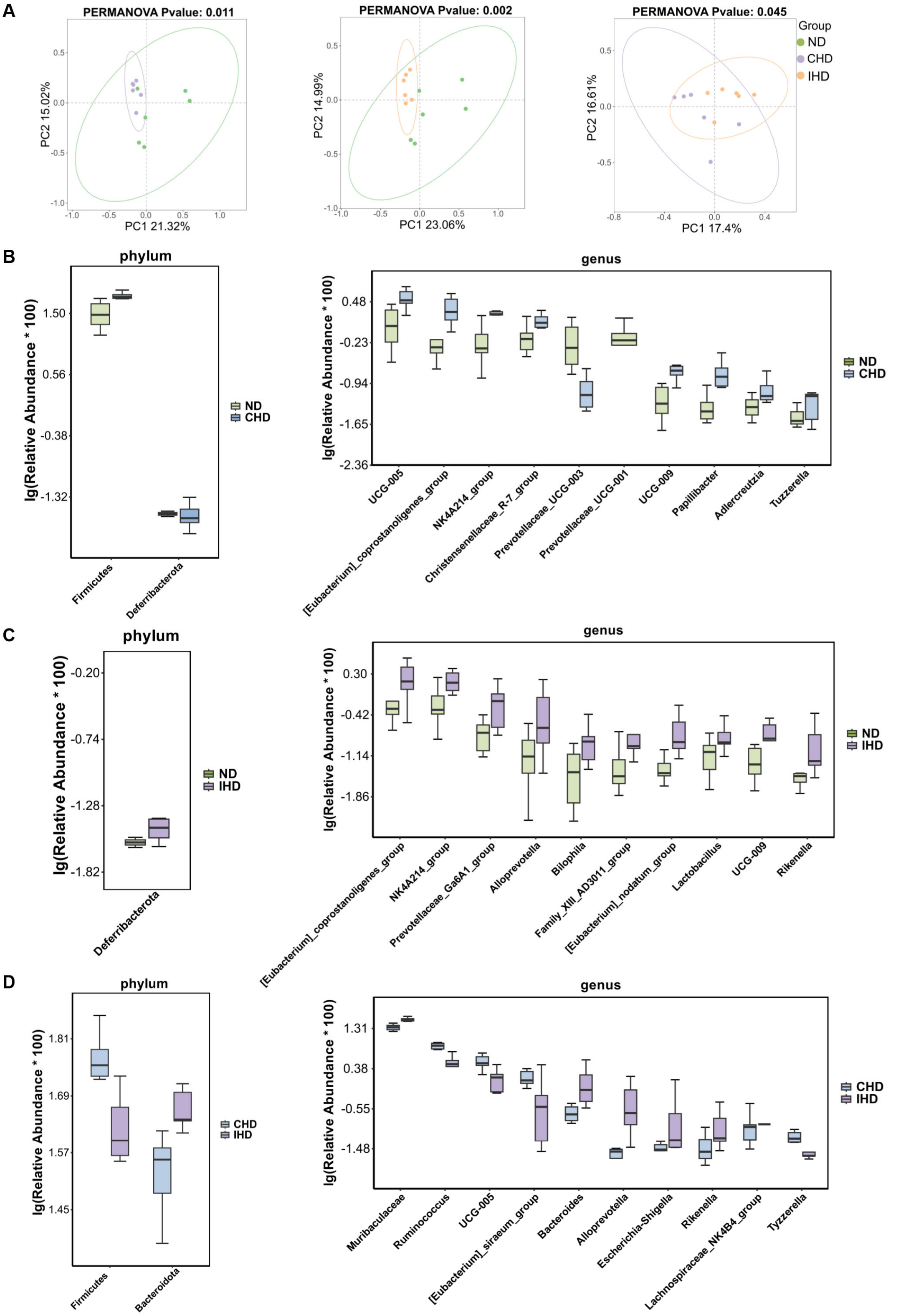

Supplementary Figure S3. Differential alterations in the gut microbiome across dietary patterns. A: β-diversity comparison between groups; B-D: Differential gut microbial abundances at the phylum and genus levels among three groups.

HFHC: high-fat, high-cholesterol; ND: normal diet; CHD: continuous high-fat, high-cholesterol diet; IHD: intermittent high-fat, high-cholesterol diet.

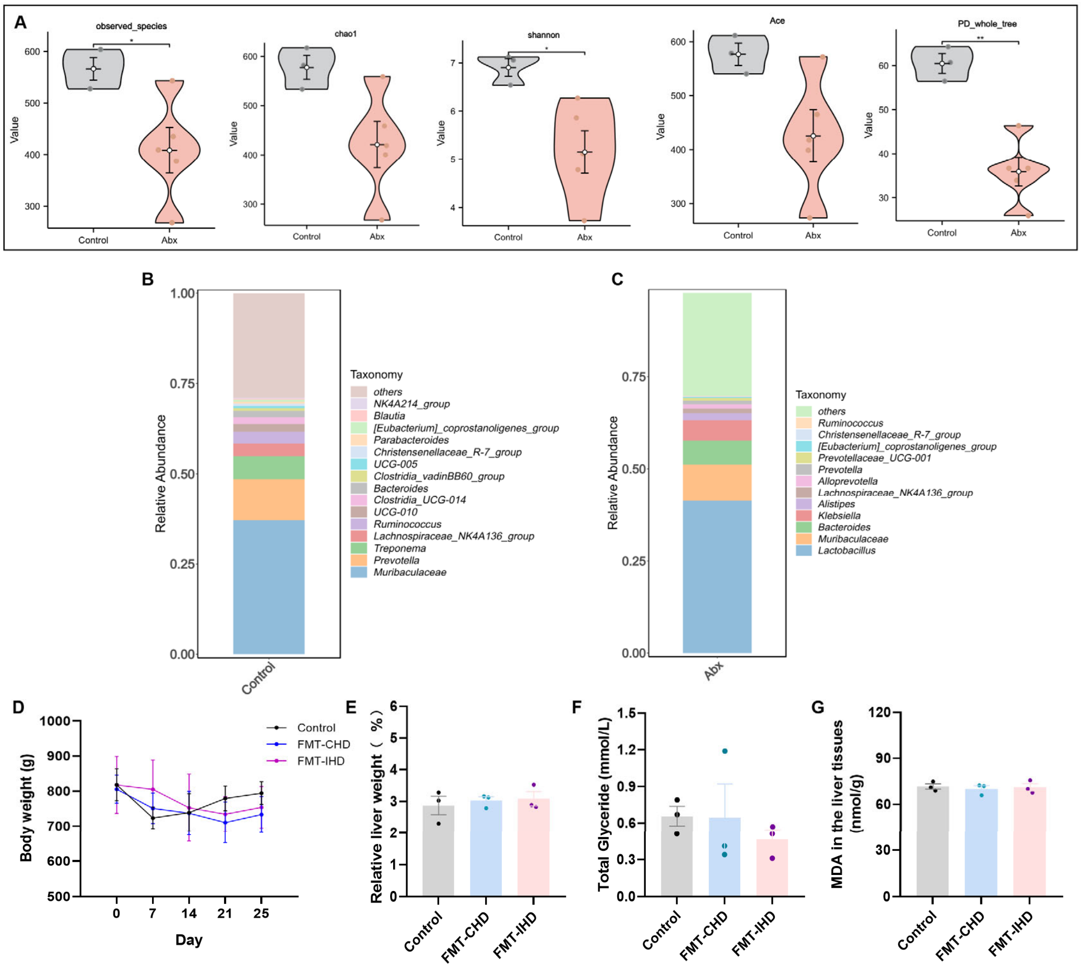

Supplementary Figure S4. Efficiency of gut microbiome elimination following antibiotic treatment in guinea pigs and physiological characteristics after fecal microbiota transplantation from donor animals fed continuous or intermittent HFHC diets.

A: α-diversity indices demonstrating gut microbiome depletion after antibiotic treatment; B and C: Comparison of gut microbiome abundance between control (B) and antibiotic-treated animals (C). D: Body weight; E: Relative liver weight; F: Serum total triglyceride levels; G : hepatic MDA levels. Data are presented as mean ± SEM in D-H. N =3 in each group.

HFHC: high-fat, high-cholesterol; ND: normal diet; CHD: continuous high-fat, high-cholesterol diet; IHD: intermittent high-fat, high-cholesterol diet; MDA: malondialdehyde.

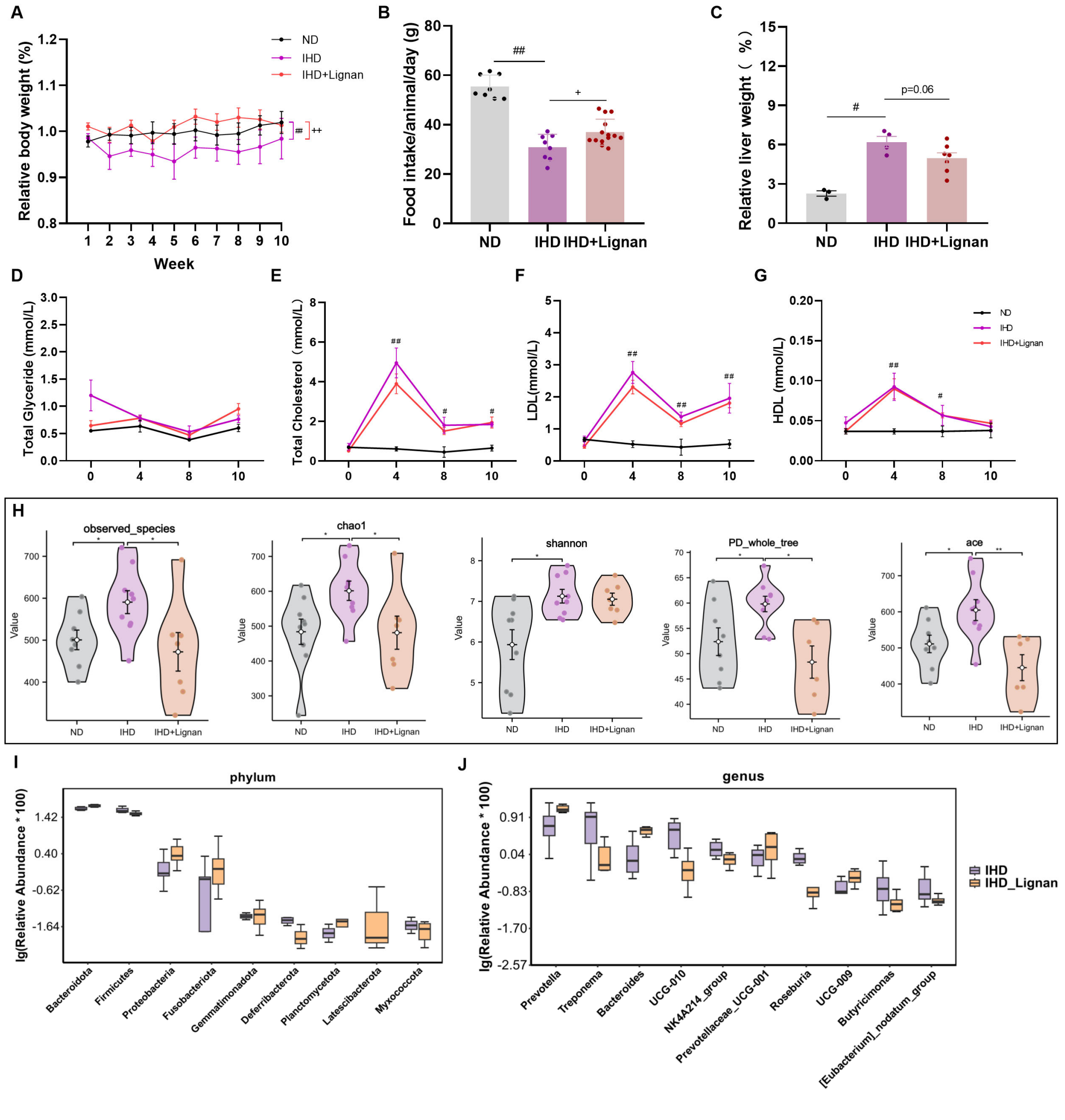

Supplementary Figure S5. Effects of flaxseed lignan treatment on physiological characteristics and gut microbiota in guinea pigs.

A: Body weight; B: Food intake; C: Relative liver weight; D-G: Blood lipid profiles including total triglycerides (D), total cholesterol (E), LDL (F), and HDL (G); H: α-Diversity indices of the gut microbiota; I-J: Differential abundance of gut microbiota at the phylum (I) and genus (J) levels between the lignan-treated group and IHD group. Data are presented as mean ± SEM in A-G. N = 3 in ND group, N=3-4 in IHD group and N=6-7 in IHD+Lignan group. #: $p < 0.05$, IHD vs ND; ##: $p < 0.01$, IHD vs ND; +: $p < 0.05$, IHD+Lignan vs IHD.

HFHC: high-fat, high-cholesterol; ND: normal diet; CHD: continuous high-fat, high-cholesterol diet; IHD: intermittent high-fat, high-cholesterol diet.

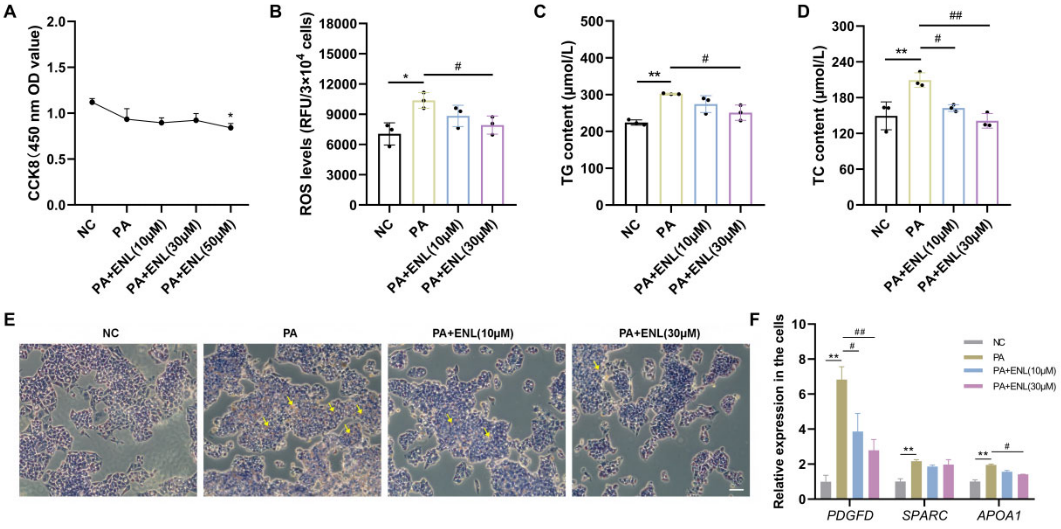

Supplementary Figure S6. In vitro effects of enterolactone on palmitic acid-challenged HepG2 cells.

A: Cell viability measured by CCK-8 assay; B: Intracellular ROS levels; C, D: Quantification of intracellular TG (C) and TC (D) contents; E: Representative images of Oil Red O staining (magnification: ×100; scale bar: 10 μm), yellow arrows indicate lipid droplets; F: qRT-PCR analysis of target gene expression.

Data are presented as mean ± SEM from three independent experiments (n = 3). NC: normal control; PA: palmitic acid (0.5 mM); ENL: enterolactone. *$p < 0.05$, **$p < 0.01$ vs. NC; #$p < 0.05$, ##$p < 0.01$ vs. PA.
